# Refining the Pneumococcal Competence Regulon by RNA Sequencing

**DOI:** 10.1128/JB.00780-18

**Published:** 2019-06-10

**Authors:** Jelle Slager, Rieza Aprianto, Jan-Willem Veening

**Affiliations:** a Molecular Genetics Group, Groningen Biomolecular Sciences and Biotechnology Institute, Center for Synthetic Biology, University of Groningen, Groningen, the Netherlands; b Department of Fundamental Microbiology, Faculty of Biology and Medicine, University of Lausanne, Lausanne, Switzerland; Ohio State University

**Keywords:** BlpR, CiaR, ComE, ComX, RNA-seq, Streptococcus pneumoniae, transcriptomics, VraR, genetic competence

## Abstract

Competence for genetic transformation allows the opportunistic human pathogen Streptococcus pneumoniae to take up exogenous DNA for incorporation into its own genome. This ability may account for the extraordinary genomic plasticity of this bacterium, leading to antigenic variation, vaccine escape, and the spread of antibiotic resistance. The competence system has been thoroughly studied, and its regulation is well understood. Additionally, over the last decade, several stress factors have been shown to trigger the competent state, leading to the activation of several stress response regulons. The arrival of next-generation sequencing techniques allowed us to update the competence regulon, the latest report on which still depended on DNA microarray technology. Enabled by the availability of an up-to-date genome annotation, including transcript boundaries, we assayed time-dependent expression of all annotated features in response to competence induction, were able to identify the affected promoters, and produced a more complete overview of the various regulons activated during the competence state. We show that 4% of all annotated genes are under direct control of competence regulators ComE and ComX, while the expression of a total of up to 17% of all genes is affected, either directly or indirectly. Among the affected genes are various small RNAs with an as-yet-unknown function. Besides the ComE and ComX regulons, we were also able to refine the CiaR, VraR (LiaR), and BlpR regulons, underlining the strength of combining transcriptome sequencing (RNA-seq) with a well-annotated genome.

**IMPORTANCE** Streptococcus pneumoniae is an opportunistic human pathogen responsible for over a million deaths every year. Although both vaccination programs and antibiotic therapies have been effective in prevention and treatment of pneumococcal infections, respectively, the sustainability of these solutions is uncertain. The pneumococcal genome is highly flexible, leading to vaccine escape and antibiotic resistance. This flexibility is predominantly facilitated by competence, a state allowing the cell to take up and integrate exogenous DNA. Thus, it is essential to obtain a detailed overview of gene expression during competence. This is stressed by the fact that administration of several classes of antibiotics can lead to competence. Previous studies on the competence regulon were performed with microarray technology and were limited to an incomplete set of known genes. Using RNA sequencing combined with an up-to-date genome annotation, we provide an updated overview of competence-regulated genes.

## Abstract (precis)

Streptococcus pneumoniae is an opportunistic human pathogen responsible for over a million deaths every year. Although both vaccination programs and antibiotic therapies have been effective in prevention and treatment of pneumococcal infections, respectively, the sustainability of these solutions is uncertain. The pneumococcal genome is highly flexible, leading to vaccine escape and antibiotic resistance. This flexibility is predominantly facilitated by competence, a state allowing the cell to take up and integrate exogenous DNA. Thus, it is essential to obtain a detailed overview of gene expression during competence. This is stressed by the fact that administration of several classes of antibiotics can lead to competence. Previous studies on the competence regulon were performed with microarray technology and were limited to an incomplete set of known genes. Using RNA sequencing combined with an up-to-date genome annotation, we provide an updated overview of competence-regulated genes.

## INTRODUCTION

Streptococcus pneumoniae (the pneumococcus) is a mostly harmless human commensal found in the nasopharynx. However, when the pneumococcus leaves the nasopharynx and ends up in other niches, it can cause severe diseases, such as sepsis, pneumonia, and meningitis (1). Especially among individuals with an underdeveloped or weakened immune system, these diseases lead to over a million deaths per year (2). Although both vaccination and antibiotic therapy have been used successfully for prevention and treatment of infections, respectively, the pneumococcus remains a threat to human health. This persistence is largely due to the remarkable genomic plasticity of the pneumococcus, allowing the acquisition of antibiotic resistance and evasion of the host immune response. Horizontal gene transfer, underlying the vast majority of such diversification strategies, is facilitated by pneumococcal competence. The competent state allows cells to take up exogenous DNA and integrate it into their own genome (i.e., transformation). During competence, various functionalities are activated, including DNA repair, bacteriocin production, and activities of several stress-response regulons (3, 4). This diversity of activated functions is relevant in light of the fact that a broad spectrum of antimicrobial compounds (causing various forms of stress) can induce competence development (5–7) through at least three distinct mechanisms: HtrA substrate competition (8, 9), *oriC*-proximal gene dosage increases (6), and chaining-mediated autocrine-like signaling (7). Other parameters that affect competence development include pH, oxygen, phosphate, and diffusibility of the growth medium (10–12). The fact that various forms of stress induce competence, leading to the activation of several stress-response regulons, has led to the hypothesis that competence in the pneumococcus may function as a general stress response mechanism (13, 14).

Among the genes activated during competence are the CiaR and VraR (LiaR) regulons. Although the underlying molecular mechanisms of activation are unknown, both regulons have been associated with cell wall damage control (3, 15). Indeed, a growth lag during competence (4) and the reduced fitness of both *ciaR* and *vraR* mutants (3, 15) indicate that competence represents a significant burden for a pneumococcal cell. It seems plausible that the production and insertion of the DNA uptake machinery (16) into the rigid cell wall have a significant impact on cell wall integrity. The CiaR regulon seems to be responsible for resolving such issues and preventing subsequent lysis (3, 17). An additional dose of competence-related cell wall stress is caused by fratricide, where competent cells kill and lyse noncompetent sister cells and members of closely related species. Specifically during competence, pneumococci produce a fratricin, CbpD, and the corresponding immunity protein, ComM (18). Secreted CbpD, aided by the action of autolysins LytA and LytC, can kill noncompetent, neighboring cells, which then release their DNA and other potentially valuable resources. Eldholm et al. showed that the VraR regulon represents a second layer of protection, beyond that represented by ComM, by which competent cells prevent CbpD-mediated lysis (15). ComM is also crucial in causing cell division arrest during competence by inhibiting initiation of division and by interfering with the activity of StkP (19).

**FIG 1:**
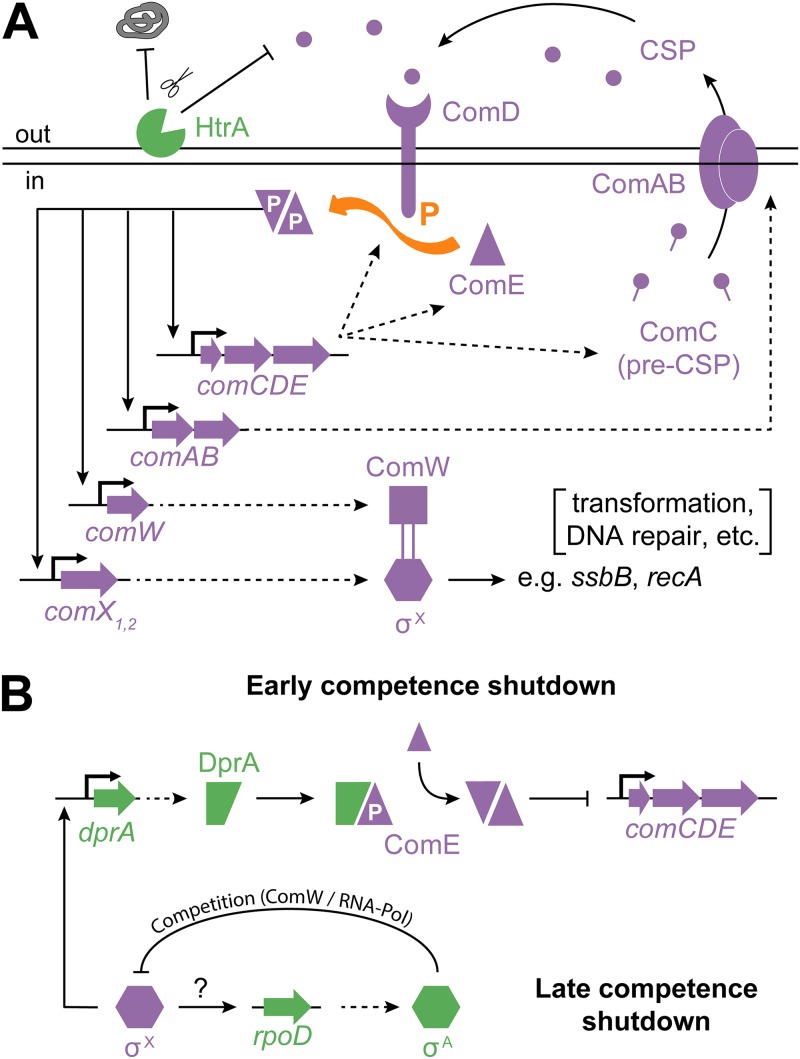
Overview of the regulatory network controlling competence in Streptococcus pneumoniae. (A) Positive-feedback loop responsible for activation of competence. HtrA-mediated degradation of CSP is relevant to the competence-inducing effect of certain antibiotics (8). (Adapted from reference 6 with permission of the publisher.) (B) Early competence is shut down through the action of late-competence protein DprA. Binding of DprA to phosphorylated ComE leads to an increase in the levels of unphosphorylated ComE dimers repressing *comCDE* expression. Late-competence shutdown is the result of competition for ComW and RNA polymerase binding between sigma factors ComX (σ^X^) and RpoD (σ^A^). Although expression of *rpoD* is strongly upregulated during competence, the underlying mechanism is unknown.

The activation of competence (Fig. 1A) depends on the action of two key transcriptional regulators, ComE and ComX. The competence regulon is divided into groups of early-competence (early-*com*) (i.e., ComE-dependent) genes and late-competence (late-*com*) (i.e., ComX-dependent) genes. Specifically, early competence involves, among others, the *comCDE* and *comAB* operons. A basal expression level of *comCDE* (20) ensures the production of the small peptide ComC, which contains a double-glycine leader and is processed and exported into the extracellular milieu by the bipartite transporter ComAB (21, 22). The resulting 17-residue matured peptide is referred to as competence-stimulating peptide (CSP) (23) and can interact with ComD, the membrane histidine kinase component of the two-component system (TCS) ComDE (24). Upon CSP binding, ComD autophosphorylates and, subsequently, transfers its phosphate group to its cognate response regulator, ComE (25). Finally, phosphorylated ComE dimerizes and binds specific recognition sequences to activate the members of the early-*com* regulon (26, 27). This regulon contains both the aforementioned *comAB* and *comCDE* operons, creating a positive-feedback loop that self-amplifies once a certain threshold of extracellular CSP is reached. Since CSP can interact with ComD on the producer cell as well as on other pneumococcal cells, competence represents a quorum-sensing system. Although cell-to-cell contact is not required for the spread of competence in a population (7, 28), it does lead to more efficient signaling (29) and to more-extensive recombination events (30).

Additional members of the early-*com* regulon are *comX1* and *comX2*, two identical genes that encode the alternative sigma factor ComX (σ^X^) (31). The rapid accumulation of ComX during early competence leads to the activation of promoters with a ComX-binding motif, resulting in the expression of the late-*com* regulon (3, 4, 32, 33). Finally, within 20 min after the initiation of competence, the process is largely shut down through a combination of different mechanisms (Fig. 1B) (34–36). Early-*com* genes are repressed by late-competence protein DprA and unphosphorylated ComE (27). As a second layer of control, sigma factor competition between ComX and RpoD (σ^A^; induced during late competence) is probably responsible for the shutdown of late-*com* genes (36).

While the backbone of this regulatory system is quite well understood, there are many other factors that complicate the matter, including the system’s sensitivity to growth medium acidity and potential small RNA (sRNA)-mediated control of ComC expression (37).

To fully understand the implications of competence activation in the pneumococcus, it is important to know which genes are (directly or indirectly) differentially expressed during competence. Several comprehensive studies, based on DNA microarray technology, have been performed to determine the competence regulon, resulting in the identification of more than 100 reported competence-associated genes (3, 4, 38). All of those studies showed a high level of agreement on a certain core regulon, but discrepancies remained. Moreover, the recent identification of early-competence protein BriC (39, 40) illustrates that the description of the competence regulon can still be refined. In order to generate a more complete and nuanced overview of the competence regulon, we first utilized data from PneumoExpress (39), a resource containing data on the pneumococcal transcriptome under various infection-relevant conditions. We used transcriptome sequencing (RNA-seq) data sets from S. pneumoniae D39V (41) cells just prior to (*t* = 0) and 3, 10, and 20 min after the addition of exogenous CSP. More importantly, our data set has higher sensitivity and precision and has a larger dynamic range than the data sets used with previous genome-wide assays of the competence regulon, which were based on DNA microarrays (42). Second, the recent reannotation of the pneumococcal genome revealed previously nonannotated protein-encoding sequences and small RNAs (41). DNA microarray studies are limited to the target sequences present on the array, and a new data set was therefore required to obtain information on the expression of these new elements. Finally, the new annotation also contains information on transcription start sites (TSSs) and terminators (41), which allows both a more accurate search of transcription regulatory motifs (e.g., ComE- or ComX-binding sites) and the integration of operon information into the interpretation of transcriptome data.

As expected, our results largely confirmed those of previous microarray-based studies and we observed distinct time-dependent expression patterns of ComE- and ComX-regulated genes. In addition, we provide an overview of the transcription start sites most likely to be responsible for the observed transcriptome changes, adding up to, among others, 15 ComE-regulated, 19 ComX-regulated, 18 CiaR-regulated, and 4 VraR-regulated operons. We identified 7 new noncoding RNAs (ncRNAs), affected by several regulators, among the differentially expressed genes, but elucidation of their role in competence requires future studies.

## RESULTS

### Competence induction disrupts the pneumococcal transcriptional landscape.

**FIG 2:**
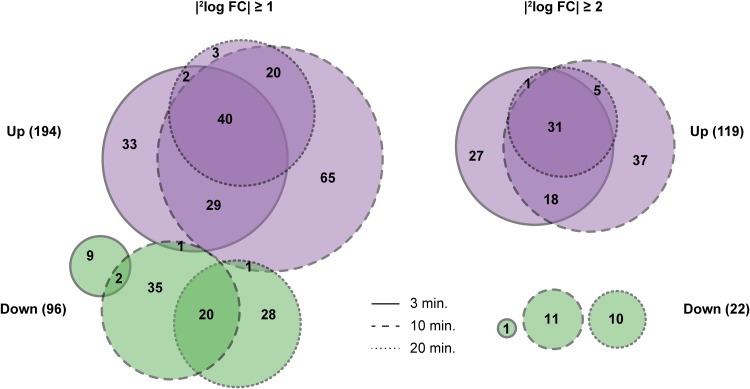
Venn diagrams of differentially expressed genes, created with http://eulerr.co. Diagrams show how many genes were significantly upregulated (purple) or downregulated (green), using a cutoff fold change value of 2 (left) or 4 (right). Differential expression results seen 3, 10, and 20 min after addition of CSP are indicated by outlines.

Differential gene expression analysis revealed that many genes (13% and 17%, corresponding to data from gene-based and promoter-based analysis, respectively) are affected by the induction of competence (Fig. 2): a total of 288 genes undergo a change in expression of more than 2-fold. Among these, 192 genes were exclusively upregulated, 94 were exclusively downregulated, and 2 genes were upregulated at one time point and downregulated at another. Using a stricter fold change cutoff value of 4-fold, 141 genes were still significantly affected, 119 of which were upregulated and 22 were downregulated. As can be seen in Fig. 2, upregulated genes tended to be affected more strongly and consistently, while not a single gene was found to be significantly downregulated at all three time points.

### Identification of ComE-, ComX-, and CiaR/VraR-regulated WGCNA clusters.

Weighted gene coexpression network analysis (WGCNA) (see Materials and Methods) of all of the genes, performed on the basis of their regularized log (rlog) expression levels across the 22 conditions included in PneumoExpress (39), yielded 36 clusters (see Table S2 in the supplemental material). Using these results, we verified whether some of the clusters corresponded to specific regulons known to be affected during competence. Indeed, one of these clusters (cluster [cl.] 29, *n* = 26) contained 20 of the 25 genes that had been reported previously to be regulated by ComE (3, 4, 38), including *briC* (SPV_0391), which was identified as a member of the competence regulon only recently (39, 40). Aggarwal et al. showed that the double-glycine peptide encoded by *briC* is conserved only in pneumococci and related streptococci and is important for late biofilm development (40). Those authors also found that BriC is secreted (in part by ComAB), but its exact mode of action has yet to be elucidated.

The five ComE-regulated genes that did not end up in this cluster include *blpA*, *blpY*, *blpZ*, and *pncP* (SPV_0472 to SPV_0475, respectively), which are part of the BlpR regulon and whose promoters are likely to have lower affinity for ComE (43, 44). The fifth off-cluster ComE-regulated gene is *ybbK* (SPV_1984). A second cluster (cl. 11, *n* = 56) contained 41 of 51 members of the reported ComX regulon (3, 4, 38), confirming the power of the WGCNA approach while simultaneously highlighting the general reliability of previous descriptions of the competence regulon. Less clearly, 13 of 32 known CiaR-regulated genes (37, 45, 46) and 5 of 14 VraR-regulated genes (15) clustered together (cl. 33, *n* = 22). The fact that genes from the CiaR and VraR regulon cluster less clearly may, in part, be explainable by the more diverse nature of their regulation. For example, the heat shock *hrcA-grpE* operon is regulated not only by VraR but also by HrcA itself, accounting for a different expression dynamic across the diverse conditions sampled for PneumoExpress (39). Additionally, the two TSSs of *tarIJ-licABC* (SPV_1127 to SPV_1123) (37) and the downregulatory effect of CiaR on the *manLMN* operon (SPV_0264 to SPV_0262) (46) may prevent clear clustering.

### Time-resolved expression profiles of several regulons during competence.

**FIG 3:**
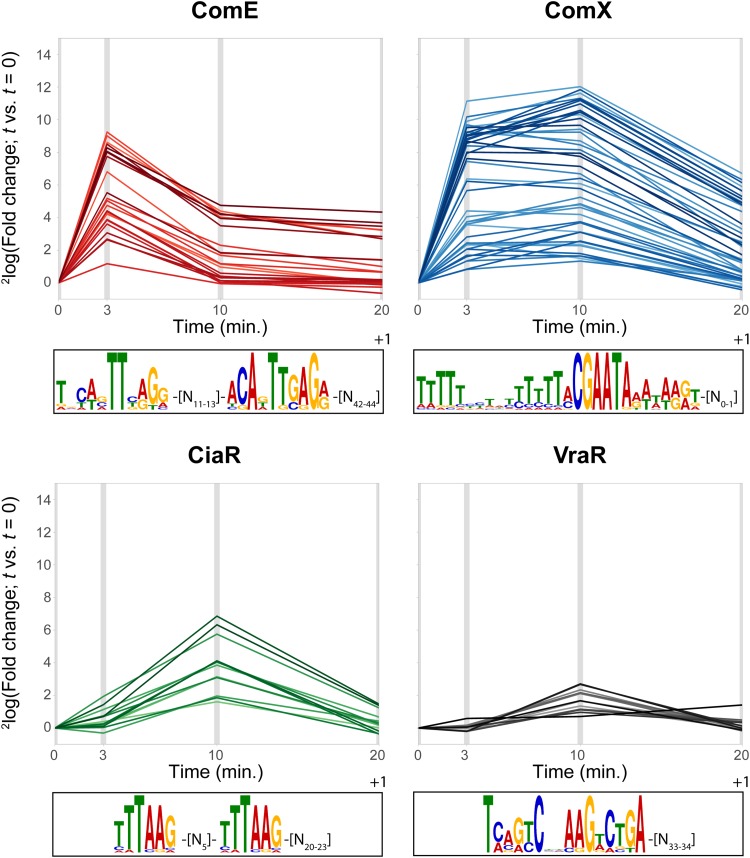
Regulons affected during competence. (Top panels) Expression profiles of genes previously reported to be ComE, ComX, CiaR, or VraR activated. Each line shows the fold changes (versus *t* = 0) for a single gene from the corresponding regulon. Except for the VraR regulon (all genes shown), only genes that fell into the appropriate WGCNA cluster (cl. 29 for ComE, cl. 11 for ComX, cl. 33 for CiaR) were included. (Bottom panels) Position weight matrices of the recognition sites for ComE, ComX, CiaR, and VraR, as determined with MEME (94), from the promoters of the HC members of the corresponding regulons (ComE, ComX, and CiaR) or from three pneumococcal and six L. lactis promoters (VraR [15]).

We visualized the typical time-resolved expression patterns of the various regulons by plotting the fold expression changes, relative to *t* = 0, of all genes that (i) were previously reported to be activated by the corresponding regulator and (ii) fell into the associated WGCNA cluster (see above). We refer to these sets of genes as “high-confidence” (HC) members of their respective regulons. It is clear from these plots that ComE-regulated genes peaked early and rapidly dropped in expression level afterward (Fig. 3, top left). This is in line with previous studies which showed that early competence is actively shut down through the action of late-competence protein DprA. By specifically binding to active, phosphorylated ComE, DprA causes a shift toward a state where regulated promoters are, instead, bound by dephosphorylated ComE, leading to a shutdown of transcription (Fig. 1B) (36, 47).

Similarly to the ComE regulon results, the level of expression of ComX-regulated genes also increased rapidly, with high fold changes after 3 min. However, the expression of these genes remained stable for a longer period of time than was seen with the ComE regulon, with most still increasing their level of expression until 10 min after CSP addition (Fig. 3, top right). The decrease in expression level that followed was, in part, indirectly linked to the shutdown of early competence, since both production and stabilization by ComW (34) of ComX depend on the activity of phosphorylated ComE. However, a recent modeling approach suggested that another shutdown mechanism was required to explain the observed rate of late-competence shutdown (36). The authors argued, convincingly, that competition between sigma factors ComX (σ^X^) and RpoD (σ^A^) for interaction with RNA polymerase and/or stabilizing factor ComW would be a suitable explanation for the discrepancies between the model data and the experimental data. Indeed, the fact that *rpoD* transcription is upregulated up to 10-fold during competence (3, 4) would make this a credible hypothesis, although *rpoD* upregulation could also simply serve to restore the expression levels of RpoD-controlled genes.

Constituting a more indirect consequence of competence induction, the CiaR-mediated response was generally weaker than and delayed in comparison to the responses seen with the ComE and ComX regulons (Fig. 3, bottom left). Interestingly, the activation of this regulon also seems to be quite transient, with a fast drop in expression from 10 to 20 min after CSP addition.

Finally, the expression profile of all reported VraR-regulated genes (15), regardless of their clustering behavior under the infection-relevant conditions, was similar to that of the CiaR regulon (Fig. 3, bottom right).

### Shared expression trends within operons allow switching from gene level analysis to promoter level analysis of transcriptional regulation.

Transcriptome studies are typically performed on a per-gene level, reporting for each individual gene whether or not it is differentially expressed under the different studied conditions. That type of information is certainly relevant in assessments of what changes occur in a cell or population under conditions of confrontation with a certain change in environment or identity. However, to find out how these changes come about, it is also interesting to consider which transcripts or, rather, which promoters have been affected. To this end, we used our previously created map of the pneumococcal transcriptional landscape (39, 41), where possible, to identify which promoters are responsible for the observed differential levels of expression of individual genes. As an example, we highlight a cluster of 22 genes (SPV_0192 to SPV_0213), encoding 21 ribosomal proteins and protein translocase subunit SecY (Table 1). Although Peterson et al. reported previously that expression of the entire operon was downregulated (4), we observed significant upregulation (just above the 2-fold cutoff) for only 10 genes and no significant effects for the rest of the operon. However, it is clear both from visual inspection and from the fact that all genes cluster together in the WGCNA analysis that the entire operon behaves as a single transcriptional unit, with modest upregulation occurring 3 min after CSP addition, followed by a drop in expression at 10 min. Therefore, regulation of a single TSS, at position 195877 [positive strand (+)], would suffice to explain the behavior of these 22 genes. Note that the observed gradient in fold changes across this operon was observed in several other operons as well (see Table S5). Although we do not fully understand this phenomenon, we speculate that differences in the rates of mRNA degradation might play a role. Since (exo)ribonucleases have a specific directionality (i.e., 5ʹ to 3ʹ or 3ʹ to 5ʹ in Gram-positive bacteria), such differences could explain the observed gradients.

**TABLE 1:** Expression trend of a 22-gene operon (SPV_0192 to SPV_0213) transcribed from TSS 195877 (+)^*a*^

Locus tag	Gene	TPM at 0 min	Log_2_ fold change at:		
			3 min	10 min	20 min
SPV_0192	*rpsJ*	4,585	0.7	0.0	−0.1
SPV_0193	*rplC*	2,992	0.9	−0.1	0.0
SPV_0194	*rplD*	2,181	0.8	−0.1	−0.3
SPV_0195	*rplW*	2,919	1.0	−0.2	0.2
SPV_0196	*rplB*	2,611	1.0	−0.2	−0.1
SPV_0197	*rpsS*	4,134	1.0	−0.5	0.1
SPV_0198	*rplV*	3,838	1.0	−0.5	0.1
SPV_0199	*rpsC*	3,158	1.0	−0.4	0.0
SPV_0200	*rplP*	4,329	1.0	−0.6	0.0
SPV_0201	*rpmC*	3,060	0.9	−0.7	0.0
SPV_0202	*rpsQ*	4,348	1.1	−0.7	−0.1
SPV_0203	*rplN*	3,640	**1.1**	−0.7	0.1
SPV_0204	*rplX*	3,917	1.1	−0.7	0.0
SPV_0205	*rplE*	2,848	**1.1**	−0.7	−0.2
SPV_0206	*rpsN*	2,717	**1.1**	−0.6	−0.3
SPV_0207	*rpsH*	4,013	**1.3**	−0.8	0.0
SPV_0208	*rplF*	4,411	**1.1**	−0.8	−0.2
SPV_0209	*rplR*	3,598	**1.1**	−0.9	−0.2
SPV_0210	*rpsE*	3,477	**1.1**	−0.9	−0.2
SPV_0211	*rpmD*	4,120	**1.1**	−0.9	−0.4
SPV_0212	*rplO*	2,247	**1.2**	−0.7	−0.2
SPV_0213	*secY*	1,920	**1.0**	−0.7	−0.3

a Boldface data indicate significance (*P* < 0.001; log_2_ fold change [FC] > 1; DESeq [91]).

### Early-competence genes: the ComE and BlpR regulons.

**FIG 4:**
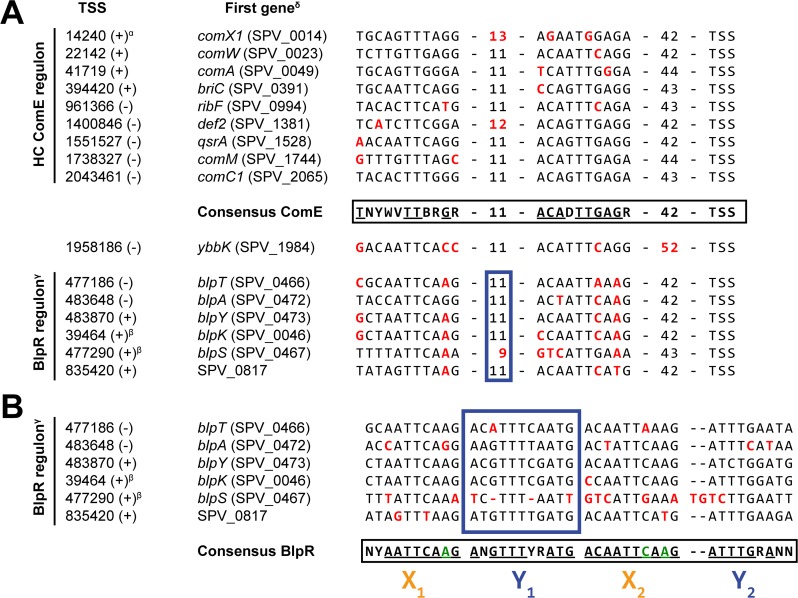
(A) ComE-binding sequences on the S. pneumoniae D39V genome. The consensus sequence was determined as described in Materials and Methods. Multiple possible nucleotides are indicated, according to IUPAC nomenclature, by R (A or G [A/G]), Y (C/T), W (A/T), B (C/G/T), D (A/G/T), V (A/C/G), or N (any). Nucleotides and spacings colored red deviate from this consensus. HC, high confidence. (B) Putative BlpR-binding site consensus. The blue box corresponds to the internal spacer indicated in panel A. The consensus sequence (IUPAC nomenclature used as described for panel A) was determined as described in Materials and Methods, where letters colored green indicate where the BlpR consensus is incompatible with the ComE consensus. A superscript alpha indicates that the promoters of *comX1* (SPV_0014) and *comX2* (SPV_1818) are identical. A superscript beta indicates that the operons were not differentially expressed in response to CSP addition. A superscript gamma indicates that the genes encoding the export machinery of signaling peptide BlpC and bacteriocins BlpK and PncW and the *blpA* and *blpB* genes were frameshifted, eliminating the regulatory positive-feedback loop of the Blp system. A superscript delta indicates the first gene annotated in both D39V and D39W (97).

We reasoned that confining the analysis of promoter regions to only those upstream of HC members (defined above) of a regulon was likely to yield a more accurate set of data representing the consensus binding site of the corresponding regulator than analysis of such a consensus on the basis of all known regulated genes, as is the common procedure. Therefore, combining transcription start site (TSS) data on these selected genes (Table S3) as reported previously (41) and known characteristics of regulatory sites (e.g., typical distances from TSS), we redefined the binding motifs for ComE, ComX, and CiaR (see Materials and Methods for details). The identified ComE-binding sequence (Fig. 3, top left; see also Fig. 4A) strongly resembles sequences described in previous reports (26, 27) and consists of two imperfect repeats. The spacing between these repeats is 11 to 13 nucleotides (nt) in length (mode = 11), while the spacing between the right motif and the TSS is 42 to 44 nt (mode = 42). Clearly, the second repeat is the more highly conserved repeat and is likely to be most important for ComE recognition. In summary, this yields the following consensus ComE-binding motif: [TNYWVTTBRGR]-[N_11_]-[ACADTTGAGR]-[N_42_]-[TSS] (Fig. 4A).

As Martin et al. previously reported (27), the internal spacing and the right arm of the binding sequence in P*_comX_* deviate from the consensus (Fig. 4A). Indeed, from our data, this deviation seemed to lead to a somewhat lower level of expression from P*_comX_* after induction (Table 2), relative to other members of the ComE regulon. This reduction is partially compensated by the presence of two copies of *comX* on the chromosome. In contrast, we found no indication that mismatches with the consensus ComE-binding sequence found in the promoter region of *comAB* resulted in lower expression of those genes (Table 2), which was suggested by Martin et al. (27) to explain an earlier observation that *comAB* transcript levels were rate-limiting in the development of competence (48). Indeed, although higher ComAB levels may indeed accelerate competence development, the fact that a duplication of *comC* (22, 25) leads to competence upregulation suggests that ComAB is not exporting CSP at maximum capacity in wild-type cells.

**TABLE 2:** ComE-regulated genes, distributed over 15 operons^*a*^

Gene	Product	TPM at 0 min	Log_2_ fold change at:			Note(s)
			3 min	10 min	20 min	
*comX1*	Competence-specific sigma factor	8	**9.1**	**3.8**	**3.1**	
*tRNA-Glu-1*	tRNA-Glu-UUC	387	**2.8**	0.1	−0.6	Secondary
*comW*	Competence positive regulator	17	**9.1**	**4.7**	**3.0**	
*purA*	Adenylosuccinate synthetase	619	**4.4**	1.0	0.0	Secondary
*ccnC*	csRNA3	155	**4.4**	1.2	0.0	Secondary; also CiaR regulon
*srf-03*	ncRNA of unknown function	39	**7.1**	2.0	1.5	
*comA*	CSP ABC transporter ATP-binding protein	21	**9.3**	**4.1**	**3.4**	
*comB*	CSP ABC transporter permease	24	**9.5**	**4.4**	**3.3**	
*briC*	Biofilm-regulating peptide	97	**4.9**	**1.7**	0.7	
*ydiL*^*b*^	Putative membrane peptidase	67	**5.3**	**2.4**	1.0	
*blpT*	BlpT protein	8	**3.9**	0.7	1.5	BlpR regulon
*blpA*^*b*^	Peptide ABC transporter permease/ATP-binding protein	2	**5.1**	**2.1**	**2.5**	BlpR regulon
*blpB*^*b*^	Peptide ABC transporter permease	3	**4.5**	**2.2**	**2.4**	BlpR regulon
*blpC*	Peptide pheromone	3	4.4	2.7	1.9	BlpR regulon
*pncW*	Putative bacteriocin	13	**4.0**	1.1	**2.0**	BlpR regulon
*blpY*	Bacteriocin immunity protein	17	**4.3**	**1.4**	**2.2**	BlpR regulon
*blpZ*	Immunity protein	13	**4.3**	1.5	**1.9**	BlpR regulon
*pncP*	Putative protease	14	**4.2**	**1.3**	**1.9**	BlpR regulon
*SPV_2249*	Hypothetical protein	50	1.2	0.3	0.8	BlpR regulon
*SPV_0817*	CAAX amino terminal protease family protein	45	**1.3**	0.2	0.4	BlpR regulon
*ribF*	FMN adenylyltransferase/riboflavin kinase	147	**5.2**	**1.2**	0.7	
*def2*	Peptide deformylase	177	**3.7**	0.0	−0.1	
*SPV_1380*	Cell shape-determining protein	276	**3.9**	0.1	0.0	Secondary
*SPV_1379*	Hypothetical protein	183	**3.3**	0.0	−0.1	Secondary
*yaaA*	UPF0246 protein	85	**1.2**	0.0	−0.3	Secondary
*qsrA*	ABC transporter ATP-binding protein-Na^+^ export	223	**4.5**	0.6	0.2	Secondary
*qsrB*	ABC transporter permease-Na^+^ export	216	**4.5**	0.4	−0.2	Secondary
*srf-22*	ncRNA of unknown function	54	**4.1**	0.2	−0.7	Secondary
*comM*	Immunity factor	9	**8.0**	**4.4**	**2.8**	
*tsaE*	tRNA processing protein	280	**3.1**	0.3	0.2	Secondary
*SPV_1742*	Acetyltransferase	319	**2.7**	0.3	0.2	Secondary
*lytR*	LytR-CpsA-Psr family protein	355	**2.8**	0.1	0.1	Secondary
*comX2*	Competence-specific sigma factor	8	**9.1**	**3.8**	**3.1**	
*tRNA-Glu-3*	tRNA-Glu-UUC	387	**2.8**	0.1	−0.6	Secondary
*ybbK*	Putative membrane protease subunit	795	**2.1**	0.6	0.6	TSS too far from ComE-binding site
*comC1*	Competence-stimulating peptide precursor	36	**8.8**	**5.1**	**4.6**	
*comD*	Two-component system sensor histidine kinase	27	**8.4**	**4.3**	**3.8**	
*comE*	Two-component system response regulator	38	**8.3**	**4.0**	**3.5**	
*tRNA-Glu-5*	tRNA-Glu-UUC	63	**5.8**	**1.9**	1.5	Secondary
*tRNA-Asn-2*	tRNA-Asn-GUU	181	**5.1**	0.8	0.4	Secondary

a The 15 operons are indicated by the eight blocks of data highlighted in gray and the seven blocks of data highlighted in white. Members of the BlpR regulon are included as indicated in the Note(s) column. “Secondary” indicates either read-through after incomplete termination or the influence of an additional TSS. For complete supplemental information, including TSS positions, see Table S5. Boldface data indicate significance (*P* < 0.001; log_2_ FC > 1; DESeq [91]). FMN, flavin mononucleotide; ncRNA, noncoding RNA.

b Pseudogene.

As reported before, the *comAB* genes are preceded by a BOX element (49), an imperfectly repeated DNA element occurring 127 times in the D39V genome (41). Interestingly, the BOX element is located downstream of the ComE-regulated TSS and is therefore part of the *comAB* transcript. It was shown by Knutsen et al. that the BOX element upstream of *comAB* is important for the fine-tuning of competence (50), but the underlying mechanism is unknown. We previously detected several RNA fragments that terminated between the BOX element and the start codon of *comA*, leading us to annotate it as an ncRNA, *srf-03* (41). Although some BOX elements were reported to contain putative protein-encoding sequences, we did not find any uninterrupted coding sequence in this specific case. It seems unlikely that the BOX element functions as an RNA switch, since *srf-03* and *comAB* displayed the same time-dependent expression patterns, with relatively low expression at *t* = 0 (Table 2). Since removing this BOX element from P*_comA_* leads to reduced *comAB* expression (50), it is tempting to speculate that the prematurely terminated transcript corresponding to *srf-03* plays a role in competence regulation. However, a similar effect on transcription was observed when the BOX element in front of *qsrAB* (also ComE regulated) was removed (50) and we did not find any evidence for premature termination between that BOX element and the start of *qsrA*.

Analysis of all upregulated promoters resulted in the detection of five additional operons putatively regulated by ComE (the complete proposed ComE regulon is listed in Table 2), including *ybbK*, a known early-*com* gene. The weaker induction of this gene and the fact that its expression did not cluster with that of other early-*com* genes can be explained by the fact that the ComE site is located 10 nt too far from the TSS, compared to a canonical ComE-regulated gene (Fig. 4A).

Three other ComE-induced operons (*blpT*, *blpABC*, and *pncW-blpYZ-pncP*) are known to be part of the BlpR regulon, and their activation is the result of cross talk, where ComE can recognize the binding sites of BlpR but with lower efficiency (43, 44). Indeed, close inspection of the corresponding promoter regions shows marked differences with those of other ComE-regulated genes, consistently deviating from the consensus ComE-binding site at specific positions (Fig. 4A). The same discrepancies were observed in the promoter regions of *blpK* and, to a lesser extent, *blpSRH*, the two remaining *blp* operons. These operons were not differentially expressed during competence, probably due to the poorer resemblance to the ComE-binding consensus. Additionally, both *blpK* and *blpSRH* are constitutively expressed, such that any minor inducing effect by ComE would be negligible. A multiple-sequence alignment of the promoter regions of the five known *blp* operons in strain D39V revealed a conserved sequence very similar to, but slightly more extended than, that of the putative BlpR-binding site postulated by de Saizieu et al. (51). The binding site reported here can be seen as the following imperfect tandem 19- to 21-bp repeat sequence: [NYAATTCAAGANGTTTYRATG]-[ACAATTCAAG(NN)ATTTGRANN]-[N_33_]-[TSS]. More specifically, the region can be written as X_1_-Y_1_-X_2_-Y_2_, where X (resembling the ComE-binding site) and Y are 10 and 9 to 11 bp in length, respectively, having a highly conserved “TT” sequence (or a TTT sequence in Y) at their centers (Fig. 4B). Interestingly, the promoter region of the final operon putatively regulated by ComE, SPV_2249 to SPV_0817, resembles the putative BlpR recognition site (Fig. 4B) and we speculate that these genes are actually part of the BlpR regulon. This idea is supported by the very modest levels of induction (2.3-fold and 2.5-fold, respectively) of this operon during competence. Additionally, SPV_0817 encodes a probable CAAX protease (41) that could be speculated to be involved in immunity against self-produced bacteriocins (52, 53).

Finally, only one feature from the WGCNA cluster associated with ComE regulation remained that could not be directly linked to a ComE-binding site. This feature, a pseudogene (SPV_2414), is part of an ISSpn*7* insertion sequence (IS) (54) and represents a truncated version of the gene encoding the corresponding transposase. Since the D39V genome contains eight additional sites with ≥95% sequence identity to this insertion sequence, clearly undermining mapping fidelity, and since no significant differential expression was observed at any competence time point, we ruled out SPV_2414 as a member of the ComE regulon.

### Late-competence genes: the ComX regulon.

**FIG 5:**
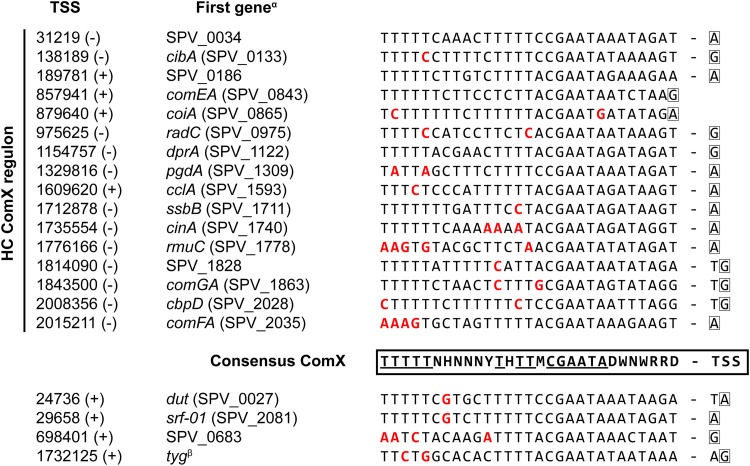
ComX-binding sequences on the S. pneumoniae D39V genome. The consensus sequence was determined as described in Materials and Methods. Multiple possible nucleotides are indicated, according to IUPAC nomenclature, by R (A/G), Y (C/T), W (A/T), M (A/C), D (A/G/T), H (A/C/T), or N (any). HC, high confidence. A superscript alpha indicates the first gene annotated in both D39V and D39W (97), if available. A superscript beta indicates the *tyg* gene described by Campbell et al. (32). Its TSS was detected in PneumoBrowse (41), but no coding DNA sequence (CDS) or ncRNA has been reported (41, 59).

Directly following the strong, ComE-mediated increase in *comX* expression, the late-competence regulon is activated. On the basis of the promoter sequences of HC members of the late-*com* regulon (Fig. 5; see also Table S3), the ComX recognition sequence was reevaluated. Not surprisingly, the identified motif (Fig. 3, top right) did contain a near-perfect match (TMCGAATA) to the previously reported 8-nucleotide consensus sequence (3, 32, 33):. However, our analysis showed that the region relevant to ComX binding is likely much wider than that; with a thymine-rich stretch upstream and a (less extensively conserved) adenine-rich stretch downstream of the reported 8 nucleotides, the actual recognition site is extended to 20 to 30 bp. In summary, this yields the following consensus ComX-binding motif: [TTTTTNHNNNYTHTTMCGAATADWNWRRD]-[TSS] (where the underlined sequence represents the previously reported consensus sequence) (Fig. 5).

In addition to the 16 HC ComX-regulated operons, we identified three promoters containing the motif reported here (Table 3). First, SPV_0027 to SPV_0030, an operon encoding, among others, a dUTP pyrophosphatase (*dut*) and DNA repair protein RadA (*radA*), was already previously reported to be part of the late-*com* regulon (3, 4) but was not found to cluster with the HC ComX regulon. A secondary TSS, 11 nucleotides downstream of the ComX-regulated TSS, might be responsible for the lower correlation with other ComX-regulated genes. Similarly, a second previously reported late-*com* gene, SPV_0683, might be under the control of a secondary, as-yet-unidentified TSS, besides the ComX-activated TSS reported here (Table 3), a speculation supported by its relatively high expression level prior to CSP addition and sequencing coverage observed in PneumoBrowse (41). A third ComX-binding site (SPV_0033) was found downstream of *prs1* and immediately upstream of a novel ncRNA, *srf-01* (SPV_2081), which we identified recently (41). While this addition to the known competence regulon seemed interesting at first, the partial overlap between the ncRNA and a nearby IS element (containing pseudogene SPV_0034) led us to question the functionality of this novel element (see Fig. S1 in the supplemental material). Additionally, another pseudogene (SPV_2082) was located on the other side of the IS element and was also found to be under the control of ComX (Table 3). A multiple-genome alignment of several pneumococcal strains (not shown) revealed that the ComX-binding element downstream of *prsA1* (i.e., *prs1*) was conserved in, among other strains, S. pneumoniae INV200 (GenBank accession number FQ312029.1) but was followed in that strain by a set of pseudogenes (Fig. S1). A BLASTX search showed that the two pseudogenes were probably derived from a protein-encoding gene mostly annotated as encoding a recombination-promoting nuclease or transposase. Interestingly, this gene was highly similar to SPV_2082 and to an additional pseudogene, SPV_2340, located elsewhere on the D39V chromosome and also ComX regulated. We speculate that the presence of a repeat unit of the pneumococcus (RUP) (55) upstream of SPV_2082, in combination with the action of IS elements, might have enabled several events of duplication and/or reorganization of the SPV_2082 locus. While these findings suggest that, in pneumococcal strains with an intact copy of this gene, it might be relevant to transformation and horizontal gene transfer, we do not expect *srf-01* (or pseudogenes SPV_2082 and SPV_2340, for that matter) to have a role in competence.

**TABLE 3:** ComX-regulated genes, distributed over 19 operons^*a*^

Gene	Product	TPM at 0 min	Log_2_ fold change at:			Note
			3 min	10 min	20 min	
*dut*	Deoxyuridine 5ʹ-triphosphate nucleotidohydrolase	78	**2.7**	**1.5**	−0.2	
*SPV_0028*	Hypothetical protein	94	**2.6**	**1.6**	−0.2	
*radA*	DNA repair protein	96	**2.5**	**2.4**	−0.1	
*SPV_0030*	Carbonic anhydrase	388	0.8	**1.1**	0.1	Secondary
*srf-01*	ncRNA of unknown function	71	**3.7**	**3.3**	0.9	Putative pseudogene
*SPV_2082*^*b*^	Hypothetical protein	22	**6.6**	**6.3**	**2.6**	
*SPV_0034*^*b*^	IS*1167* transposase	3	3.6	**3.7**	1.1	
*cibA*	Two-peptide bacteriocin peptide	8	**12.0**	**12.9**	**7.4**	
*cibB*	Two-peptide bacteriocin peptide	11	**10.2**	**11.0**	**5.5**	
*cibC*	CibAB immunity factor	10	**10.6**	**12.3**	**6.5**	
*SPV_2121*	Hypothetical protein	124	**4.6**	**4.4**	0.6	
*SPV_0186*	Competence-damage induced protein	283	**2.4**	**2.3**	0.1	Secondary
*SPV_0683*	Hypothetical protein	136	**4.2**	**4.9**	0.8	
*comEA*	Late-competence DNA receptor	4	**9.8**	**9.2**	**3.7**	
*comEC*	Late-competence DNA transporter	3	**9.5**	**9.8**	**5.0**	
*SPV_2256*	Hypothetical protein	156	**4.0**	**4.8**	**1.4**	Secondary
*SPV_2257*^*b*^	ABC transporter ATP-binding protein	91	**3.7**	**5.0**	**1.3**	Secondary
*SPV_0846*	Hypothetical protein	68	**3.9**	**5.4**	**1.5**	Secondary
*coiA*	Competence protein	1	**8.2**	**7.4**	1.2	
*pepF1*	Oligoendopeptidase F	252	**1.3**	**1.2**	0.0	Secondary
*SPV_0867*	*O*-Methyltransferase family protein	136	**1.4**	**1.5**	0.3	Secondary
*radC*	DNA repair protein	3	**9.8**	**9.8**	2.6	
*dprA*	DNA protecting protein	6	**10.1**	**8.8**	**4.6**	
*SPV_2340*^*b*^	Hypothetical protein	222	**2.5**	**2.6**	0.2	Secondary
*pgdA*	Peptidoglycan *N*-acetylglucosamine deacetylase	397	**1.4**	**1.7**	0.1	Secondary
*SPV_1308*	Oxidoreductase of aldo/keto reductase family, subgroup 1	182	0.9	**1.4**	0.3	Secondary
*cclA*	Type IV prepilin peptidase	2	**9.3**	**9.0**	**3.7**	
*ssbB*	Single-stranded DNA-binding protein	9	**10.7**	**11.9**	**6.8**	
*cinA*	ADP-ribose pyrophosphatase/nicotinamide-nucleotide amidase	78	**5.8**	**6.5**	**2.5**	
*recA*	DNA recombination/repair protein	391	**3.0**	**3.8**	0.7	Secondary
*dinF*	MATE efflux family protein	288	**2.2**	**3.8**	0.4	Secondary
*lytA*	Autolysin/*N*-acetylmuramoyl-l-alanine amidase	703	**1.4**	**3.2**	0.4	Secondary
*tyg*^*c*^	NA	<1	6.7	6.8	4.2	
*rmuC*	DNA recombination protein	314	**2.1**	**1.8**	−0.3	
*yhaM*	3ʹ→5ʹ exoribonuclease	312	**1.7**	**1.7**	−0.4	
*SPV_1828*	Hypothetical protein	142	**6.5**	**6.0**	**1.4**	
*nadC*	Quinolinate phosphoribosyltransferase	72	**1.7**	**3.2**	0.3	Secondary
*SPV_1825*^*b*^	IS*630*-Spn1 transposase	162	0.5	**1.7**	0.0	Secondary
*SPV_1824*	ABC transporter, permease	34	0.9	**2.7**	0.2	Secondary
*comGA*	Late-competence protein	14	**9.4**	**10.4**	**5.1**	
*comGB*	Late-competence protein	8	**9.4**	**10.8**	**5.4**	
*comGC*	Late-competence protein	6	**9.3**	**11.2**	**5.3**	
*comGD*	Late-competence protein	4	**10.1**	**12.1**	**6.3**	
*comGE*	Late-competence protein	3	**10.3**	**12.3**	**6.5**	
*comGF*	Late-competence protein	2	**9.9**	**12.2**	**6.5**	
*comGG*	Late-competence protein	8	**8.7**	**11.0**	**5.5**	
*SPV_2427*^*b*^	*S*-Adenosylmethionine-dependent methyltransferase	2	**10.3**	**13.0**	**7.2**	
*srf-29*	ncRNA of unknown function	3	**9.3**	**8.8**	3.0	
*cbpD*	Choline-binding protein D	6	**9.9**	**10.0**	**3.8**	
*SPV_2027*	Cytoplasmic thiamin-binding component of thiamin ABC transporter	89	1.0	**1.2**	−0.7	Secondary
*thiX*	Thiamin ABC transporter transmembrane component	88	0.8	**1.2**	**−1.1**	Secondary
*thiY*	Thiamin ABC transporter substrate-binding component	134	0.8	**0.9**	**−1.1**	Secondary
*thiZ*	Thiamin ABC transporter ATPase component	128	0.6	**1.0**	**−1.2**	Secondary
*comFA*	DNA transporter ATPase	3	**9.0**	**8.1**	**2.9**	
*comFC*	Phosphoribosyltransferase domain protein	3	**8.6**	**8.5**	2.7	
*hpf*	Ribosome hibernation promotion factor	624	0.9	**1.4**	−0.3	Secondary

a The 19 operons are indicated by the 9 blocks of data in a gray background and the 10 blocks of data in a white background. “Secondary” indicates either read-through after incomplete termination or the influence of an additional TSS. For complete supplemental information, including TSS positions, see Table S5. Boldface data indicate significance (*P* < 0.001; |log_2_ FC| > 1; DESeq [91]). NA, data not available.

b Pseudogene.

c The *tyg* TSS was previously found to be ComX regulated (32). An artificial 250-nucleotide transcript starting at this TSS was added to the annotation file to allow differential expression analysis.

A second ncRNA, *srf-29*, is located upstream of and partially overlaps *cbpD* (SPV_2028), a known late-*com* gene. A high-confidence terminator (41) inside the coding region of *cbpD* marks the 3ʹ end of *srf-29*. It is not clear whether *srf-29* represents an uncharacterized RNA switch regulating *cbpD* expression, produces a functional sRNA, or simply is an artifact produced by a premature terminator [see PneumoBrowse coordinates 2008356 to 2008242 (−)].

**FIG 6:**
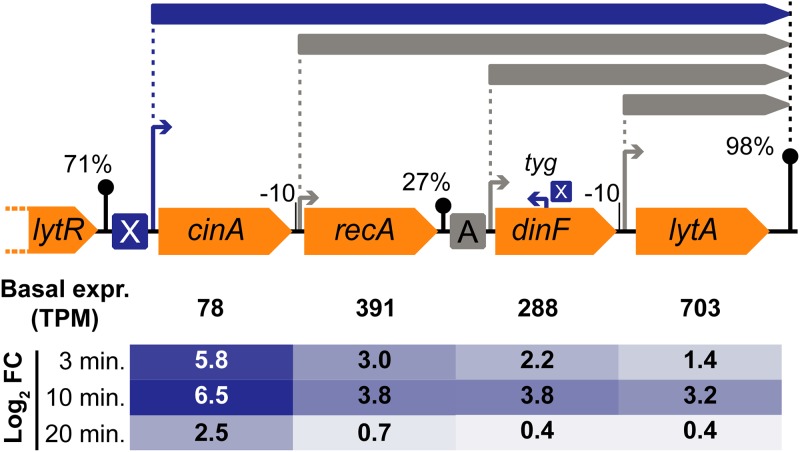
(Top) Overview of the complex *cinA-recA-dinF-lytA* operon, with an imperfect internal terminator and TSSs upstream of each gene, leading to the presence of four overlapping operons. The TSS upstream of *cinA* is preceded by a ComX-binding site, and addition of CSP indeed affected expression of all four genes in the operon. (Bottom) Log_2_ fold change relative to *t* = 0 (i.e., basal expression [expr.]). However, the effect size decreased with every gene, due to differences in basal expression from the internal TSSs. Additionally, a ComX-regulated TSS was found inside and antisense to *dinF*, giving rise to the hypothetical transcript *tyg* (32), with an unknown 3ʹ end.

Since TSS and terminator information permits a promoter-based interpretation of our data, we observed examples of complex operon structures wherein TSSs or imperfect terminators inside the operon can lead to differences in the expression levels of different genes in the same operon (41, 56, 57). A striking example is the *cinA-recA-dinF-lytA* operon (SPV_1740 to SPV_1737), shown in Fig. 6, which is under the control of ComX, with only an inefficient (27%) terminator between *recA* and *dinF*. However, the presence of three internal TSSs, upstream of *recA*, *dinF*, and *lytA*, respectively, led to very different basal expression levels at *t* = 0 (Table 3). Due to these differences, the extent to which competence induction affects the expression of the four genes decreases from the 5ʹ end to the 3ʹ end of the operon (Fig. 6). Finally, Campbell et al. identified a transcription start site inside and antisense to *dinF* that was induced during competence and provisionally named the associated hypothetical gene *tyg* (Fig. 6) (32). Although not discussed by Campbell and coworkers, both Håvarstein (58) and Claverys and Martin (59) argued that the peculiar positioning of the *tyg* TSS represents reason for doubts regarding the functionality of any transcript produced. However, as Claverys and Martin concede, the possibility cannot be ruled out that *tyg* has a role in mRNA stability of the *cinA-recA-dinF-lytA* operon. With Cappable-seq (60), we did indeed detect a transcription start site (41), accompanied by a consensus ComX recognition site (Fig. 6). Since we did not detect a clearly demarcated associated transcript, we artificially annotated a 250-nucleotide-long transcript, starting at the *tyg* TSS, to allow differential expression analysis. The time-dependent trend of expression of this transcript during competence seemed to follow that of other late-*com* genes (39). However, the extremely low expression level detected prior to (and even after) CSP addition precluded any further statistical analysis regarding differential expression or clustering. We reiterate that TSS and terminator data regarding other competence-specific operons can be retrieved from PneumoBrowse (41).

The expression pattern of 44 of the 56 members of the ComX-associated WGCNA cluster (cl. 11) can now be linked to a ComX-regulated TSS. Bearing in mind that the clustering was performed based on expression under all 22 infection-relevant conditions studied in PneumoExpress (39), only five other cluster members (SPV_0553, SPV_0957 to SPV_0959, and SPV_2317) showed an expression pattern similar to that seen with ComX-regulated genes specifically under competence conditions. While the TSS for SPV_0553 has not been determined, this gene is surrounded by two repeat units of the pneumococcus (55) and one BOX element (49) and nothing resembling a ComX-binding site was found near it. Second, SPV_2317 represents a novel ncRNA (*srf-19*), potentially an RNA switch, that is preceded by a predicted RpoD site, rather than a ComX site. The last cluster member, operon SPV_0957 to SPV_0959, contains *rpoD* (SPV_0958). In light of the proposed role of RpoD in the shutdown of late competence (see above), it would be interesting if its upregulation was directly induced by ComX. However, analysis of the promoter region of the operon yielded no indication of a ComX-binding site and the mechanism of *rpoD* induction in competence continues to elude us.

### The CiaR regulon is induced during competence and contains a novel noncoding RNA.

Besides early-*com* genes and late-*com* genes, terminology reserved for the ComE and ComX regulons, respectively, many other genes are more indirectly affected by the addition of CSP. Although a small portion of these genes can already be seen to be affected after 3 min, here we refer to all of these genes collectively as “delayed,” as these changes occur at least after the activation of the ComE regulon and most likely also downstream of the ComX regulon. Among the delayed genes are nearly all members of the CiaR regulon (Table 4), and promoter analysis of the HC members of the CiaR regulon (Table S3; see also Fig. S2) identified the following CiaR recognition site as previously reported (37, 45, 46): [TTTAAG]-[N_5_]-[TTTAAG]-[N_22_]-[TSS] (Fig. 3, bottom left). Analysis of other affected promoters turned up three additional monocistronic operons (*ccnC*, SPV_0098, and SPV_0775), all of which have already previously been reported to be regulated by CiaR. While SPV_0098 is expressed from two different TSSs (45) and *ccnC* (SPV_2078) expression is affected by transcriptional read-through from the upstream ComE-regulated *comW* operon, it is not clear why SPV_0775 does not cluster with other CiaR-regulated genes.

**TABLE 4:** CiaR-regulated genes, distributed over 18 operons^*a*^

Gene	Product	TPM at 0 min	Log_2_ fold change at:			Note
			3 min	10 min	20 min	
*ccnC*	csRNA3	155	**4.4**	1.2	0.0	Also ComE regulon
*SPV_0098*	Glycosyltransferase, group 2 family	218	−0.5	**1.0**	0.3	
*ccnE*	csRNA5	32	0.6	0.8	0.2	
*ccnA*	csRNA1	27	−2.0	−1.4	−1.0	
*ccnB*	csRNA2	2	−0.6	2.8	−1.5	
*ccnD*	csRNA4	25	0.4	**1.9**	0.0	
*manL*	Mannose-specific PTS IIAB components	3,379	0.0	−*3.3*	−1.0	Also CcpA-binding site
*manM*	Mannose-specific PTS IIC component	3,296	0.1	−*3.1*	−*1.1*	
*manN*	Mannose-specific PTS IID component	4,750	0.2	−*3.3*	−*1.0*	
*rimP*	Bacterial ribosome SSU maturation protein	228	−0.2	1.0	0.8	CiaR-binding motif on opposite strand
*nusA*	Transcription termination/antitermination protein	206	0.1	**1.0**	0.9	
*SPV_0480*	Putative transcription termination protein	117	0.5	**1.2**	**1.1**	
*SPV_0481*	L7Ae family ribosomal protein	103	0.6	**1.4**	**1.4**	
*infB*	Translation initiation factor 2	277	0.8	0.7	0.9	
*rbfA*	Ribosome-binding factor A	194	**1.0**	0.5	0.8	
*ciaR*	Two-component system response regulator	225	0.2	**3.2**	0.3	
*ciaH*	Two-component system sensor histidine kinase	164	0.3	**3.2**	0.2	
*SPV_0775*	Acetyltransferase	28	0.4	**4.8**	1.0	
*prsA*	Putative parvulin type peptidyl-prolyl isomerase	267	**1.3**	**3.9**	0.6	Potentially also ComX regulon
*SPV_0913*	Extracellular protein	55	**2.0**	**5.9**	**1.3**	
*rlmCD*	23S rRNA [uracil(1939)-C(5)]-methyltransferase	38	−0.4	**2.0**	0.4	Secondary
*tarI*^*b*^	Ribitol-5-phosphate cytidylyltransferase	308	0.6	0.4	0.2	
*tarJ*^*b*^	Ribulose-5-phosphate reductase	362	0.4	0.3	0.1	
*licA*^*b*^	Choline kinase	319	0.6	0.2	0.1	
*licB*^*b*^	Choline permease	397	0.6	0.0	−0.1	
*licC*^*b*^	Cholinephosphate cytidylyltransferase	422	0.8	0.0	0.0	
*srf-21*	ncRNA of unknown function	325	0.7	**3.2**	0.3	
*axe1*	Acetyl xylan esterase 1/cephalosporin-C deacetylase	58	0.8	**4.2**	0.2	Secondary
*SPV_1769*	Membrane protein	497	0.0	**1.9**	−0.4	
*malQ*	4-Alpha-glucanotransferase (amylomaltase)	69	0.1	**4.2**	−0.2	
*malP*	Maltodextrin phosphorylase	83	0.2	**4.2**	−0.4	
*dltX*	d-Alanyl-lipoteichoic acid biosynthesis protein	277	−0.6	**1.9**	0.2	CiaR-binding motif on opposite strand
*dltA*	d-Alanine-poly(phosphoribitol) ligase subunit 1	392	−0.3	**1.6**	0.1	
*dltB*	d-Alanyl transfer protein	322	0.0	**1.6**	0.0	
*dltC*	d-Alanine-poly(phosphoribitol) ligase subunit 2	429	0.0	**1.5**	0.1	
*dltD*	Poly(glycerophosphate chain) d-alanine transfer protein	326	0.3	**1.5**	0.0	
*htrA*	Serine protease	49	**1.5**	**7.0**	**1.5**	
*parB*	Chromosome partitioning protein	57	0.9	**6.5**	**1.4**	Secondary

a The 18 operons are indicated by the 9 blocks of data in a gray background and the 9 blocks of data in a white background. “Secondary” indicates either read-through after incomplete termination or the influence of an additional TSS. For complete supplemental information, including TSS positions, see Table S5. Boldface data indicate significance (*P* < 0.001; |log_2_ FC| > 1; DESeq [91]). PTS, phosphotransferase system; SSU, small subunit.

b The indicated operon has two different detected TSSs. The TSS at 1159217 (−) is under the control of CiaR.

**FIG 7:**
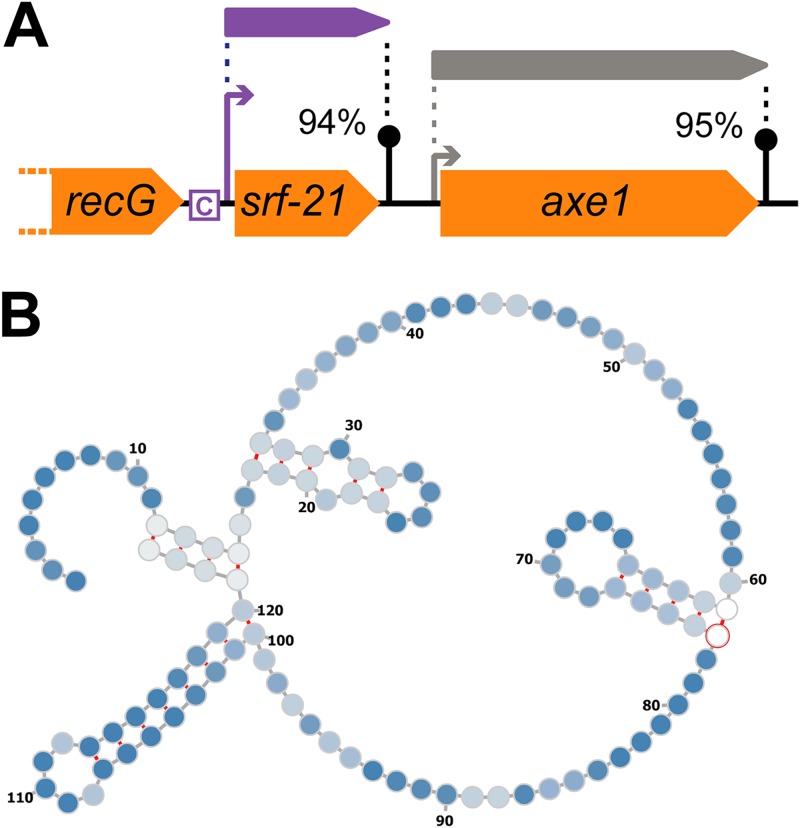
Noncoding RNA *srf-21* (SPV_2378) is part of the CiaR regulon. (A) Overview of the genomic context of *srf-21*. CiaR-dependent upregulation of downstream gene *axe1* might be due to read-through from *srf-21*. The CiaR-binding sequence is indicated by a boxed “C.” (B) MFE secondary structure of *srf-21*, as predicted by RNAfold (63).

Since CiaR-binding sites were found on the opposite strand for *dltXABCD* (SPV_2006 to SPV_2002; upregulated) and *manLMN* (downregulated), we speculate that the operon containing, among others, *rimP*, *infB*, *nusA*, and *rbfA* (SPV_0478 to SPV_0483), which encode several proteins involved in translation, is also regulated by CiaR (Fig. S2). Intriguingly, another new member of the CiaR regulon is *srf-21* (SPV_2378), a novel, uncharacterized noncoding RNA (41). The TSS from which this ncRNA is expressed was already part of the reported CiaR regulon but was linked to the overexpression of the downstream *axe1* gene (SPV_1506). Inspection of the transcriptional layout of the region (Fig. 7A) showed that *srf-21* and *axe1* are separated by a relatively efficient terminator and a second TSS. Nonetheless, *axe1* overexpression might still be attributable to read-through from *srf-21*. We did not find any similar ncRNAs in the RFAM and BSRD databases (61, 62), and, since *axe1* is expressed from its own TSS, it seems unlikely that *srf-21* functions as an RNA switch. Preliminary minimum free energy (MFE) secondary structure prediction performed with RNAfold (63) and target prediction performed with TargetRNA2 (64) did not provide us with any clear hints with regard to the function of this ncRNA. First, the predicted MFE structure (Fig. 7B) might represent only a transient conformation, since it makes up less than 1% of the modelled ensemble. Second, sRNA target prediction produced many potential targets (Table S4). Some candidate regions are less likely to be targeted, because they are located more than 20 nucleotides upstream of the start codon or even upstream of the TSS, ruling out a possible interaction between *srf-21* and the transcript in question. However, future work will be necessary to reveal whether any of the remaining genes (e.g., *queT*, *pezA*, and *cps2H*) are regulated by *srf-21* or, indeed, whether this ncRNA might have a completely different mode of action.

It is noteworthy that four reported promoters of the CiaR regulon were not found to be significantly affected during competence. First, the *tarIJ-licABC* operon is under the control of two TSSs and is thereby apparently less sensitive to CiaR control. Finally, three of five *cia*-dependent small RNAs (csRNAs), described by Halfmann et al. (37) did not appear to be significantly affected (Table 4). Note, however, that the quality of the statistical data on these short transcripts is rather poor and that the current data can neither support nor disprove their upregulation in competence. However, we do believe that the data presented by Halfmann et al. regarding CiaR-regulation seem perfectly convincing and the promoter regions of each of the five csRNAs contain a clear match with the consensus CiaR-binding site (Fig. S2).

### Other known regulons affected during competence.

Regardless of whether or not competence should be regarded as a stress response mechanism in itself, it is clear that the activation of competence, at least indirectly, leads to a multifactorial stress response. Still unknown in previous descriptions of the competence regulon, the VraR (LiaR) regulon has been described by Eldholm et al. to be activated in response to competence-induced cell wall damage (15). On the basis of three pneumococcal promoters and six Lactococcus lactis promoters (Table S3; see also Fig. S3), we rebuilt the consensus motif described by Eldholm et al. (Fig. 3, bottom right) and observed that all of these motifs are located 32 to 34 nucleotides upstream of the corresponding TSS. In contrast, we were able to confirm the reported presence of a VraR-binding site upstream of *hrcA* (15), but this site is 81 nucleotides removed from its target TSS. However, the fact that this promoter region also carries two HrcA-binding sequences could account for this difference in spacing. Finally, we suggest that SPV_1057 (spr1080 in R6) and SPV_1160 (spr1183) are not regulated by VraR, in contrast to the report by Eldholm and coworkers. First, both of these genes lacked a detected TSS and neither was found to be differentially expressed in our study. Second, the reported recognition site for SPV_1057 is actually located downstream of the gene, inside a repeat region (ISSpn*7* element). Finally, as recognized by Eldholm et al., SPV_1160 represents a 5ʹ-truncated version of a gene putatively encoding the ATP-binding component of an ABC transporter. These observations, combined with the fact that the reported VraR-binding site is located only 24 nucleotides upstream of the annotated start of SPV_1160, led us to conclude that SPV_1160, like SPV_1057, is not regulated by VraR. This limits the VraR regulon to 15 genes, distributed over 4 operons (Table 5). Eldholm et al. showed that specifically CbpK (PcpC; SPV_0357) and a putative phage shock protein (SPV_0803) are important in preventing competence-mediated lysis, forming an additional layer of protection besides ComM (15).

**TABLE 5:** Competence-induced genes that are part of the VraR, CtsR, and/or HrcA regulons^*a*^

Gene	Product	TPM at 0 min	Log_2_ fold change at:			Regulon(s)	Note
			3 min	10 min	20 min		
*spxA2*	Transcriptional regulator SpxA2	772	0.0	**1.4**	0.0	VraR	
*SPV_0179*	Hypothetical protein	163	−0.2	**1.7**	0.1	VraR	Secondary
*clpL*	Clp protease ATP-binding subunit ClpL	24	−0.2	**6.6**	**3.7**	CtsR	
*vraT*	Cell wall-active antibiotic response protein VraT	62	0.1	**2.4**	−0.1	VraR	
*vraS*	Two-component system sensor histidine kinase VraS	52	0.0	**2.2**	0.0	VraR	
*vraR*	Two-component transcriptional regulator VraR	48	0.1	**2.2**	−0.3	VraR	
*alkD*^*b*^	DNA alkylation repair enzyme AlkD	43	0.0	**2.2**	0.0	VraR	
*SPV_0355*	Hypothetical protein	118	−0.2	**1.2**	0.5	VraR	Secondary
*cbpG*^*a*^	Choline-binding protein CbpG	156	−0.2	**1.1**	0.4	VraR	Secondary
*cbpK*	Choline-binding protein CbpK	151	0.1	**1.0**	0.3	VraR	Secondary
*hrcA*	Heat-inducible transcription repressor HrcA	650	−0.2	**2.7**	0.1	VraR, HrcA	
*grpE*	Heat shock protein GrpE	600	−0.3	**2.7**	−0.1	VraR, HrcA	
*dnaK*	Chaperone protein DnaK	1,005	−0.6	**2.5**	−0.2	VraR, HrcA	
*SPV_2171*	Hypothetical protein	679	−0.8	**2.3**	−0.1	VraR, HrcA	
*dnaJ*	Chaperone protein DnaJ	551	−0.6	**2.1**	−0.1	VraR, HrcA	
*clpE*	Clp protease ATP-binding subunit ClpE	168	0.2	**1.4**	0.9	CtsR	
*SPV_0803*	Putative phage shock protein C	316	0.1	**1.7**	−0.1	VraR, HrcA	
*groES*	Heat shock protein 60 family cochaperone GroES	1,099	0.5	**2.5**	0.6	CtsR, HrcA	
*groEL*	Heat shock protein 60 family chaperone GroEL	1,146	0.3	**2.2**	0.6	CtsR, HrcA	
*ctsR*	Transcriptional regulator CtsR	162	−0.2	**1.3**	**1.2**	CtsR	
*clpC*	Clp protease ATP-binding subunit ClpC	257	−0.1	1.0	0.9	CtsR	
*SPV_2021*	Hypothetical protein	220	0.3	**1.1**	**1.0**	CtsR?	
*SPV_2020*	Two-component system response regulator	167	0.1	0.9	**1.1**	CtsR?	
*SPV_2019*	Two-component system sensor histidine kinase	86	0.4	**1.1**	**1.2**	CtsR?	

a The different operons are indicated by blocks of data in a gray background and blocks of data in a white background. “Secondary” indicates either read-through after incomplete termination or the influence of an additional TSS. For complete supplemental information, including TSS positions, see Table S5. Boldface data indicate significance (*P* < 0.001; |log_2_ FC| > 1; DESeq [91]).

b Pseudogene.

Besides VraR and CiaR, the well-characterized HrcA (3, 65) and CtsR (3, 66) regulons are also activated in competent cells, as previously reported (3). The only addition to be made here regarding the HrcA regulon is the annotation of a gene encoding a protein of unknown function (SPV_2171) that had not been annotated in D39 or R6 strains previously. This gene is located between *dnaK* (SPV_0460) and *dnaJ* (SPV_0461) and is therefore regulated by both VraR and HrcA (Table 5). Expression of almost the entire CtsR regulon was found to be upregulated. The observed upregulation was below the employed cutoff only for *clpP* (SPV_0650), possibly because its basal expression level (*t* = 0) is 4-fold to 40-fold higher than that of other *clp* genes. Finally, the upregulation of an uncharacterized two-component regulatory system (SPV_2020 and SPV_2019), with unknown consequences, could be attributed to transcriptional read-through from the upstream *ctsR-clpC* operon.

Together, CtsR and HrcA account for the overproduction of several subunits of the Clp protease system and of several chaperone proteins (e.g., DnaK, DnaJ, and GroES-GroEL) during competence (Table 5). Both of these protein functions are aimed at the reduction of stress caused by misfolded proteins and might be required to ensure proper folding of the many competence proteins.

Two other regulons seemed overrepresented in the set of differentially expressed genes (*P* < 10^−4^, hypergeometric test). First, transcription of all six genes predicted to be regulated by GntR (SPV_1524), on the basis of homology to Streptococcus pyogenes Spy_1285 (67), was found to be upregulated 10 and 20 min after addition of CSP. These six genes are distributed over two operons (SPV_0686 to SPV_0688 and SPV_1524 to SPV_1526). Second, expression of a significant number (*n* = 31) of RpoD-regulated genes was downregulated, mostly after 10 and 20 min, which may readily be explained by the competition for RNA polymerase of RpoD (σ^A^) with the alternative sigma factor ComX (σ^X^).

### Other differentially expressed genes.

A total of 367 genes (i.e., 17% of all annotated genes) were either found to be differentially expressed or, at least, to be under the control of a TSS that appears to be differentially regulated at some point during competence (Table S5). The response of a large portion (204 genes) of these can be ascribed to the action of one of the transcriptional regulators discussed above. While 56% of the members of the latter group displayed a maximum absolute change in expression of more than 4-fold, only 29 of the remaining 163 genes (16%), distributed over 14 operons, met the same criterion. These data show that the bulk of strong induction or repression can be explained by the activity of a small set of regulators. Among the 29 strongly differentially expressed genes with no known regulators, our data confirmed the upregulation of *rpoD*, in line with the role that RpoD might play in late-competence shutdown (36).

Functional analysis did not reveal any gene ontology (GO) or KEGG classes overrepresented among upregulated genes. Only classes related to ribosomes and translation seemed overrepresented, but the realization that all affected genes from these classes were part of a single operon (shown in Table 1) led us to discard them due to lack of evidence (see Materials and Methods). Similarly, most of the potential hits among downregulated genes were discarded. Only classes related to thiamine metabolism (GO 0009228, KEGG ko00730) remained. The five genes in question are distributed over four operons: *adk* (SPV_0214), *thiM-1-thiE-1* (SPV_0623 and SPV_0624), *thiD* (SPV_0632), and *sufS* (SPV_0764). Two of these four operons are regulated by a TPP riboswitch, an RNA element that, when bound to thiamine pyrophosphate (TPP), prevents transcription of the downstream operon (68). We suspect, therefore, that the temporary growth lag accompanying competence development (3) leads to a transient accumulation of TPP, which then represses transcription of operons under the control of a TPP riboswitch. Indeed, both of the other D39V operons led by a TPP riboswitch showed a similar expression trend 10 to 20 min after competence induction. First, *ykoEDC-tenA-thiW-thiM-2-thiE-2* (SPV_0625 to SPV_0631) is already minimally expressed prior to CSP addition, preventing significant downregulation (not shown). The second operon, SPV_2027-*thiXYZ* (SPV_2027 to SPV_2024), was excluded from gene enrichment analysis since it was part of the ComX regulon (Table 3). Since the hypothesized accumulation of TPP seems to occur with a delay, relative to the activation of the ComX regulon, expression of these genes is first upregulated (3 to 10 min) and then downregulated (20 min) even relative to the basal expression level.

### Comparison to previous reports of the competence regulon.

Finally, we compared our findings with those reported from previous, microarray-based studies by Peterson et al. (4) and Dagkessamanskaia et al. (3). Although both reports give a remarkably complete overview, our approach allowed us to refine and nuance the description of the competence regulon even further (Table S5). The higher levels of sensitivity and accuracy of Illumina sequencing, the improved genome annotation, and the application of a promoter-based (rather than gene-based) analysis allowed us to expand the set of genes identified as under direct control of ComE and ComX to 40 and 55 genes, respectively (combined, 4% of all genes). In particular, several genes with putative BlpR-binding sites (Table 2) and, therefore, a generally weaker response were missing in previous reports. Additionally, the previously reported *briC* operon (40) is now included in the ComE regulon and we confirmed that, while undetected by Dagkessamanskaia et al., *ybbK* and *def2* (early) and *radC* (late) are indeed part of the *com* regulon (Tables 2 and 3). Remaining discrepancies could be explained either by transcriptional read-through or by the absence of certain elements (e.g., ncRNAs) from the TIGR4 and R6 genome annotation files used by Peterson et al. and Dagkessamanskaia et al., respectively.

Not surprisingly, larger discrepancies were found for genes displaying delayed differential expression, since their fold changes are (mostly) considerably smaller and therefore more sensitive to technical variation and differences in experimental conditions (e.g., growth medium, pH, or time of induction). In contrast with the previous studies, we identified nearly the entire CiaR regulon as differentially expressed in our data, confirming the higher sensitivity of RNA-seq than of microarray-based technology.

## DISCUSSION

Competence for genetic transformation is defined as a state in which a bacterial cell can take up exogenous DNA and incorporate it into its own genome, either in the form of a plasmid or via homologous recombination. Since the very first demonstrations of bacterial transformation were provided by Griffith and Avery et al. (69, 70) in S. pneumoniae, the pneumococcal competence system was widely studied as soon as the required tools became available. Therefore, much knowledge has been assembled about how this state is regulated, which environmental triggers affect its development, and what downstream consequences it has. Indeed, several studies have been performed to compile a comprehensive list of all competence-regulated pneumococcal genes. The most recent of these studies (3, 4), although of very high quality and invaluable to the research field, date from nearly 15 years ago. Since then, the fields of transcriptome analysis and genome sequencing and annotation have been revolutionized by second-generation (e.g., Illumina) and third-generation (e.g., PacBio) sequencing techniques. Therefore, we have analyzed Illumina-based RNA-seq data (39), using the recently sequenced and deeply annotated S. pneumoniae D39V strain (41), to refine the previously reported pneumococcal competence regulon. Additionally, rather than just reporting affected individual genes, we used previously determined transcript boundaries (TSSs and terminators [41]) to identify the affected promoters, which may be used to gain more insights into the regulatory processes at work during competence.

In short, we report that ComE directly regulates 15 early-*com* transcripts (40 genes), including 4 transcripts (10 genes) that are part of the BlpR regulon (Table 2; see also Fig. 4). Alternative sigma factor ComX (σ^X^) was found to control 19 late-*com* transcripts (55 genes), in addition to the previously described *tyg* TSS, inside and antisense to *dinF* (Table 3). We should note that four genes from the early-*com* and late-*com* regulons (e.g., *blpC*) did not meet the requirements with respect to fold change and/or statistical cutoff values but were part of operons that were clearly regulated. For each of these genes, the observed expression trends correlated with those of other operon members.

Our data confirmed that, as shown in the previous studies (3, 4), the activation of the early-competence and late-competence regulons indirectly resulted in the activation of several other regulons, most of which are implicated in pneumococcal stress response. First, in the newly compiled CiaR regulon (18 transcripts, 38 genes), based on the work of Halfmann et al. (37), 4 operons were not found to be significantly affected (Table 4). Other affected regulons were those under the control of VraR (LiaR), HrcA, CtsR, and GntR (see Table S5 in the supplemental material). Additionally, transcription of genes led by a TPP riboswitch was downregulated, suggesting a transient increase in intracellular thiamine pyrophosphate levels in competent cells.

Expression of many other genes, including *rpoD*, is upregulated or downregulated through unknown mechanisms. In total, approximately 140 transcripts (containing 367 genes, 17% of all genes) undergo some extent of differential expression. For several reasons (e.g., fold changes near cutoff, expression from multiple TSSs, or poor statistics due to low expression), 79 genes from these operons did not individually meet the detection criteria, leaving 288 differentially expressed genes (13%) following the traditional, gene-based analysis approach (Fig. 2). Among the affected genes are several small, noncoding transcripts. Some of these ncRNAs, like the CiaR-activated csRNAs (see below), have been characterized, and we showed that *srf-01* is unlikely to be functional. For others, e.g., *srf-03* (upstream of *comAB*) and *srf-21* (upstream of *axe1*), future work is required to determine their role, if any, during competence.

Given the fact that so many different functionalities are activated during competence, including stress response systems such as the Clp protease and several chaperone proteins, Claverys et al. proposed to refer to the system more neutrally as “X-state” (for ComX). We would argue, however, that the primary response to a high extracellular CSP level is the activation of the ComE and ComX regulons, which mostly encode proteins relevant to transformation. First, the expression of fratricin CbpD (SPV_2028), along with immunity protein ComM (SPV_1744), allows the lysis of neighboring noncompetent cells, which may offer access to both nutrients and DNA (71, 72). The upregulation of the gene encoding autolysin LytA (*lytA*) would fit in nicely here, since the simultaneous deletion of *lytC* and *lytA* abolishes competence-induced lysis completely (71). However, basal-level *lytA* expression was reported to be already sufficient for the observed lysis rate (73). Next, the DNA uptake machinery comes into play, involving many proteins encoded by the late-*com* genes, as reviewed by Claverys et al. (16); most of ComGC to ComGG (SPV_1861 to SPV_1857), ComEA/C (SPV_0843 and SPV_0844), and ComFA (SPV_2035) are, together with constitutively expressed endonuclease EndA (SPV_1762) and through the action of prepilin peptidase CclA (SPV_1593) and proteins ComGA/B (SPV_1863 and SPV_1862), assembled into a pilus-like structure (74). The importation of DNA is followed by DNA processing and recombination and involves DNA protection protein DprA (SPV_1122), recombinase RecA (SPV_1739), single-stranded DNA (ssDNA)-binding SsbB (SPV_1711), and other late-competence proteins (75–78). For the reasons discussed above, combined with the fact that the vast majority of other differentially expressed genes display a more modest change in expression (Table S5), we decided to continue calling the system competence for genetic transformation. We do, however, agree that the downstream effects of competence development should not be ignored, as discussed below.

The genome of Streptococcus pneumoniae D39V contains 13 two-component systems (TCSs) for regulation (41, 79). Interestingly, the regulons of two TCSs (ComDE and BlpRH) are activated during competence, while the regulons of another two TCSs (CiaRH and VraRS) are activated shortly after. Expression of a fifth, uncharacterized TCS (SPV_2020 and SPV_2019) is also slightly upregulated, possibly due to transcriptional read-through from the *ctsR-clpC* operon. The additional activation of stress-related regulons of HrcA and CtsR gives support to the hypothesis, as proposed by Prudhomme at al. (5), that competence activation serves as a general stress response in the pneumococcus, which lacks a LexA-mediated SOS response such as is common in many other bacteria. The activation of competence in response to various types of stress (5–8) provides even more relevance to this idea. On the other hand, Dagkessamanskaia et al. showed that a deletion of the CiaR regulon caused an extended growth lag, as well as stronger activation of the HrcA and CtsR regulons, after competence induction (3). Both observations are in line with the notion that the development of competence is accompanied by a significant burden to the cell. To our knowledge, the exact mechanism underlying CiaR-mediated prevention of autolysis is unknown. However, Halfmann et al. showed that the deletion of CiaR-induced noncoding csRNA4 and csRNA5 led to an enhanced-lysis phenotype (37). Furthermore, d-alanylation of lipoteichoic acids by the DltXABCD proteins (45) might contribute to cell wall integrity during or after the transient block in cell division that accompanies competence (19). Finally, HtrA can play a role in the cleaning up of potential misfolded or excess proteins that cause stress to the pneumococcus (9).

We consider it likely that the main benefit of the activation of the CiaR and other stress response regulons during competence is that of dealing with the stress invoked by competence itself, by building the large membrane- and cell wall-protruding DNA uptake machinery and the production of cell wall hydrolases such as the fratricin CbpD. It is not unthinkable, however, that the activation of the many different stress response regulons renders it beneficial to a pneumococcal cell to become competent even under specific stressful conditions that do not require DNA uptake or recombination machineries. Whether or not such conditions played a role in the (co)evolution of competence and downstream processes is open to speculation. In this respect, it is interesting that the production of competence-induced bacteriocins was found to be important to prevent “intruder” pneumococci from colonizing (80).

The apparent severity of the stress imposed on a competent cell emphasizes the need to shut down competence after a short transformation-permissive time window. In addition to the known role of DprA in early-competence shutdown, Weyder et al. proposed that the upregulation of *rpoD* is responsible for the (less efficient) shut-down of late competence (36). Related to this, RpoD-regulated genes are, to some extent, overrepresented among the downregulated genes during competence. While this explains the need for upregulation of *rpoD*, the underlying mechanism is still unknown. Other aspects that might play a role in the shutting down of competence are the CiaR-mediated upregulation of HtrA, which has been shown to degrade extracellular CSP (9), and the recently discovered CiaR-regulated noncoding csRNAs (*ccnA* to *ccnE*) (37, 81–83), which were shown to repress ComC translation in an additive fashion.

Finally, although several of the activated regulons are quite well understood, a large portion of affected genes are still differentially expressed through unknown mechanisms. It seems plausible that the expression of many of these genes is due to the sudden and severe shift in metabolic state. For example, the higher translational demands present during competence could lead to the upregulation of genes encoding ribosomal proteins (Table 1). Similarly, the hypothetical transient increase in TPP concentrations, leading to riboswitch-mediated downregulation of four operons, could be accompanied by the accumulation or depletion of other, unknown metabolites, with potential transcriptional consequences.

## MATERIALS AND METHODS

The samples studied here represent a subset of the data set presented in PneumoExpress (samples C+Y; CSP, 3 min; CSP, 10 min; CSP, 20 min [39]), and detailed procedures regarding bacterial growth, RNA isolation, and sequencing and mapping of reads are reported there. The key points of these methods are summarized below.

### Culturing and harvesting of S. pneumoniae D39V.

Eight tubes with 2 ml C+Y medium (pH 6.8; nonpermissive for natural competence [39]) without antibiotics were each inoculated with wild-type S. pneumoniae D39V cells (initial optical density at 600 nm [OD_600_], ∼0.004) and incubated at 37°C (standing culture in ambient air). When the cultures reached an OD_600_ of 0.05, two cultures were harvested for RNA isolation (*t* = 0). To the other six, 100 ng/ml synthetic competence-stimulating peptide (CSP-1), purchased from GenScript (Piscataway, NJ), was added. Duplicate samples were harvested 3, 10, and 20 min after CSP-1 addition. Before harvesting, cultures were pretreated with a saturated ammonium sulfate solution as described before (84), to prevent protein-dependent RNA production and degradation (85 [patent]). Afterward, cells were harvested by centrifugation (20 min, 4°C, 10,000 × *g*) and cell pellets were snap-frozen with liquid nitrogen and stored at −80°C.

### Total RNA isolation, library preparation, and sequencing and mapping of reads.

RNA was isolated using phenol-chloroform extraction, followed by DNase treatment and another round of phenol-chloroform extraction (39). The quantity and quality of total RNA were estimated by the use of NanoDrop technology, while a 1% bleach gel (86) was employed to confirm the presence of rRNA bands (23S, 2.9 kbp; 16S, 1.5 kbp) and the absence of genomic DNA. Subsequently, RNA quality was again checked using chip-based capillary electrophoresis (Agilent Bioanalyzer). Stranded cDNA library preparation was performed, without depletion of rRNA, using a TruSeq stranded total RNA sample preparation kit (Illumina, USA). Sequencing was performed on an Illumina NextSeq 500 system, in 75-nucleotide single-end mode. The raw FASTQ data are accessible at https://www.ncbi.nlm.nih.gov/geo/ with accession number GSE108031 (samples B05 to B11).

After a quality check performed with FastQC v0.11.5 (87), reads were trimmed using Trimmomatic 0.36 (88). Alignment of trimmed reads to the reference S. pneumoniae D39V genome (GenBank CP027540 [41]) was performed with STAR (89).

### Read quantification and differential gene analysis.

The aligned reads were then counted (90) according to the D39V annotation file (GenBank CP027540 [41]) in a strand-specific fashion, allowing mapping to multiple sites (-M), for which fractional counts are reported (–fraction), and allowing reads to overlap multiple features (-O) to account for polycistronic operons.

Subsequently, we analyzed the libraries in R-studio (R v3.4.2). We performed differential gene expression analysis on rounded raw counts by DESeq2 (91). Normalized expression levels are presented as TPM (transcripts per million) (92) and can be found in Table S1 in the supplemental material. Genes with an absolute change of expression of more than 2-fold and a corresponding *P* value of below 0.001 were considered to be significantly differentially expressed.

When possible, PneumoBrowse (https://veeninglab.com/pneumobrowse [41]) was used to trace back differential expression levels of individual genes to specific TSSs and promoter regions. As a starting point, the operon prediction from PneumoBrowse was used to define groups of genes differentially expressed in competence. Note that strong transcriptional responses such as those observed during competence may have significant downstream effects. Even in the presence of highly efficient transcriptional terminators, which were defined to be operon boundaries in PneumoBrowse, such read-through effects may be visible. Therefore, these coexpressed groups were refined by inspection of the raw data in PneumoBrowse and in accordance with the consideration that minor read-through from a highly expressed gene can still be significant if the expression level of the downstream gene is sufficiently lower.

### Clustering and creation of position weight matrices.

Using the weighted gene coexpression network analysis (WGCNA) R software package (93), genes were clustered based on their *rlog* (regularized log) expression value (Table S2), as output by DESeq2, across all 22 infection-relevant conditions analyzed in PneumoExpress (39). We noticed that the reported members of the ComE (26, 27), ComX (3, 4, 38), and CiaR (37, 45, 46) regulons each largely ended up in specific clusters (here, clusters 29, 11, and 33 for ComE, ComX, and CiaR, respectively). Previously reported regulon members that properly clustered in these three identified main clusters, which are referred to here as “training sets” (Table S3), were used to define the recognition motifs of these three regulators, in the form of position weight matrices (PWMs) and to determine the most common distance of such a motif from the TSS. Using the MEME suite (94), we analyzed the upstream regions in each training set for enriched sequence motifs. First, since earlier work had shown slightly different consensus sequences for the two tandem ComE boxes that make up the ComE site (27), we extracted the left ComE motif (CEM_L_) by scanning the regions from 77 to 63 bp upstream and the right motif (CEM_R_) by scanning the regions from 56 to 42 bp upstream of TSSs in the training set. The ComX-binding motif (CXM) was determined from the regions 35 bp upstream to the +1 site (TSS). In building the CEM_L_, CEM_R_, and CXM PWMs, each sequence in the training set was required to have exactly one match to the motif, in the transcription direction (i.e., on the locally defined “plus” strand).

CiaR has been described to bind to a direct repeat (37), and we scanned the regions 41 to 19 bp upstream, allowing multiple hits per sequence in the training set. While some members of the CiaR regulon have binding sites on the opposite strand, no such genes were part of the training set and the CiaR-binding motif (CRM) was therefore also limited to the plus strand. Genes reported to belong to the VraR (LiaR) regulon (15) did not cluster together throughout the 22 sets of conditions, and the TSS was unknown for some of these genes. To enable extraction of a VraR-binding motif (VRM), we combined upstream regions of pneumococcal genes *spxA2* (SPV_0178), *vraT* (SPV_0350), and SPV_0803 with those of six Lactococcus lactis genes that were reported to be regulated by the close VraR homolog CesR (15, 95) as follows: llmg_0165, llmg_0169, llmg_1115, llmg_1155, llmg_1650, and llmg_2164. Cappable-seq (60) was used to identify L. lactis TSSs (S. B. van der Meulen and O. P. Kuipers, unpublished data). Importantly, we did not use the standard “0-order model of sequences” as a background model for motif discovery but instead created background models based on the corresponding regions upstream of all known TSSs in the pneumococcal genome (e.g., −35 to +1 for ComX). Additionally, we defined summary consensus sequences using IUPAC nucleotide coding. Since the CiaR-binding motif reportedly consists of two perfect repeats, we determined the consensus based on the 16 motif occurrences in the CiaR training set (8 promoter sequences). Single-base codes (A, C, G, and T) were called when 75% (rounded up) of all promoters matched. Double-base codes (R, Y, S, W, K, and M) were called when 8/9 (ComE and VraR), 15/16 (ComX), or 5/5 (BlpR) promoters matched either of the two encoded bases. Triple-base codes (B, D, H, and V) were called when all promoters matched either of the three encoded bases. Note that, due to its degenerate appearance, the *blpRS* promoter was excluded from determinations of the BlpR-binding consensus.

### Assigning putative regulons.

After creating PWMs for ComE-, ComX-, CiaR-, and VraR-binding sites, we used FIMO (96) to scan the 100 bp upstream of all known pneumococcal TSSs for matches to these motifs. Here, too, we used the appropriate background models (see above). A cutoff false-discovery rate (*q*) value of 0.01 was used for hits with ComX- and VraR-binding motifs. We defined a reliable ComE-binding site as CEM_L_-[N_11-13_]-CEM_R_, using a cutoff *P* value of 0.01 for each motif. Similarly, we defined a CiaR-binding site as CRM-[N_5-6_]-CRM. Additionally, to assign a gene cluster to a given putative regulon, we also applied a constraint to the position of the motif relative to the corresponding TSS, based on the typical spacing observed in the training sets. Thus, the allowed first nucleotide positions were as follows: −77, −76, −75, −74, or −73 for ComE; −30, −29, or −28 for ComX; −40, −39, −38, −37, or −36 for CiaR; and −51, −50, −49, −48, or −47 for VraR.

Putative binding sites for other regulatory proteins were copied from the propagated S. pneumoniae D39 regulons, as found in the RegPrecise database (67) and annotated in PneumoBrowse (41). RNA switches, annotated in D39V, were also taken into consideration as putatively responsible regulatory mechanisms.

### Functional analysis of differentially expressed genes.

Differentially expressed genes that could not be ascribed to the action of ComE, ComX, CiaR, or VraR were subjected to gene set enrichment analysis (functional analysis). For this, gene ontology and KEGG classifications (Table S6) were extracted from the GenBank file corresponding to the latest annotation of S. pneumoniae D39V (41). Additionally, predicted transcription factor binding sites were used to assign genes to their putative regulons (Table S6). A total of 448 hypergeometric tests were performed, and a Bonferroni-corrected cutoff *P* value of 0.0001 (i.e., 0.05 divided by 448) was used to determine whether certain regulons or gene ontology or KEGG classes were overrepresented among differentially expressed genes. We excluded overrepresented classes for all affected genes belonging to the same operon, since the activation of a single promoter does not confer any statistical evidence.

### Data availability.

The data analyzed here can be extracted from PneumoExpress (https://veeninglab.com/pneumoexpress-app). Raw RNA-seq data used to build PneumoExpress were deposited to the GEO repository under accession number GSE108031.

## Supplementary Material

Supplemental file 1

Supplemental file 2
