# Supplementary material for: Refining the Pneumococcal Competence Regulon by RNA Sequencing
Source: J Bacteriol. 2019 Jun 10;201(13):e00780-18. doi: 10.1128/JB.00780-18 (PMC6560143; doi:10.1128/JB.00780-18)
Supplement: Supplemental file 1 [file JB.00780-18-s0001.pdf]

Supplemental table legends

Table S1. Expression values (TPM) of all *S. pneumoniae* D39V genes.

Table S2. Expression clusters, as determined by weighted gene co-expression network analysis (WGCNA; (1)).

Table S3. Training sets used to define consensus binding motifs for ComE, ComX, CiaR and VraR. Genes in the training sets for ComE, ComX and CiaR were both previously reported members of the corresponding regulon and fell into their core expression clusters (cl. 29 for ComE, cl. 11 for ComX, cl. 33 for CiaR). For VraR, a selection of reported regulon members from *S. pneumoniae* and *L. lactis* MG1363 was used (2). TSSs in *S. pneumoniae* D39V were extracted from the annotation file accompanying PneumoBrowse. TSSs in *L. lactis* were extracted from 5'-enriched sequencing data (S.B. van der Meulen, O.P. Kuipers, unpublished). *The *comX1* and *comX2* regions are duplicates and the corresponding promoter sequence was used only once.

Table S4. Genes putatively targeted by *srf-21*, as predicted by TargetRNA2 (3). Target coordinates have been modified to match D39V, rather than old D39W annotation (4). Where possible, distance relative to the transcriptional start site is given. Interaction sites further than 20 bps upstream of the start codon or largely upstream of the TSS are shown in blue and red, respectively. The candidate region upstream of *queT* (SPV_0898; orange) is inside the reported preQ1-II leader (5).

Table S5. All differentially expressed genes in response to CSP addition. Reports in literature were used to predict putative regulation by HrcA (6), CtsR (7), GntR (From RegPrecise; (8)) and/or a PyrR RNA switch (9, 10). Purple and green cells indicate significance ($p < 0.001$, $|^2\log FC| > 1$; DESeq (11)). [α]Expression of *ccnC* is controlled by both ComE (TSS at 22142 (+)) and CiaR (TSS at 23967 (+)). [β]Operon has two detected TSSs. The TSS at 183663 (+) is under control of VraR. [γ]Expression of SPV_2027-*thiXYZ* is controlled both by ComX (TSS at 2008356 (-)) and a TPP riboswitch (TSS at 2006885 (-)).

Table S6. Gene Ontology and KEGG classifications and predicted regulons used for gene enrichment analysis, as reported in PneumoBrowse (9).

Supplemental figures

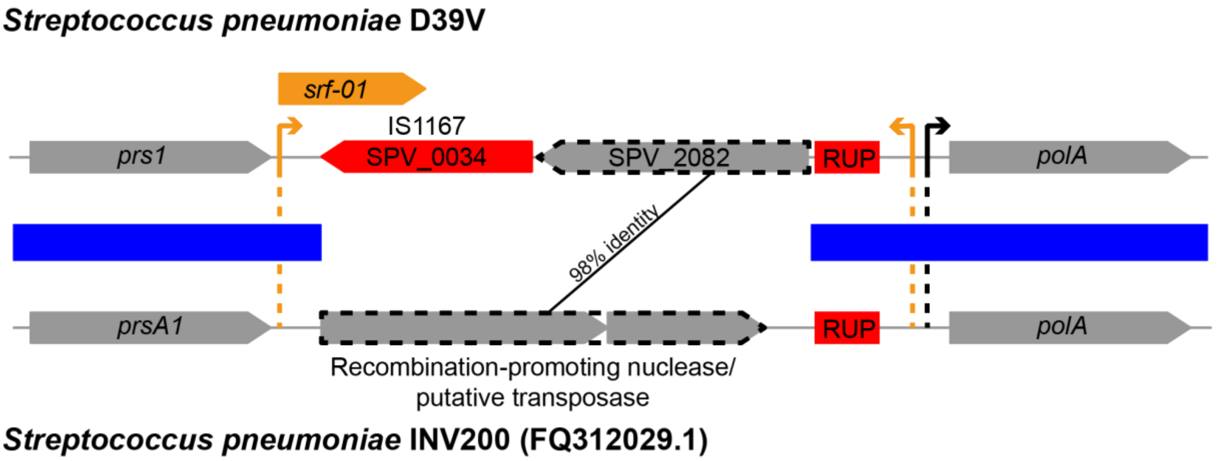

Figure S1. Comparison of *S. pneumoniae* D39V *srf-01* (SPV_2081) region and the corresponding region on the *S. pneumoniae* INV200 genome (GenBank: FQ312029.1). Blue blocks indicate conserved sequences, orange arrows indicate ComX-regulated transcription start sites, while the black arrow indicates an RpoD-regulated transcription start site. Red blocks represent repeat-containing sequences.

Group	TSS	First gene[α]	Sequence
HC CiaR regulon	234171 (+)	*ccnD* (SPV_2133)	CTTAAG - 5 - TTTAAG - 22 - A
	715262 (+)	*ciaR* (SPV_0701)	GTTGAG - 5 - TTTAAG - 22 - A
	883185 (+)	*prsA* (SPV_0868)	TTTCAG - 5 - TTTAAG - 22 - G
	1040897 (-)	SPV_0913	TATAAG - 5 - TTTAAG - 22 - G
	1528643 (-)	*srf-21* (SPV_2378)	CTTAAG - 5 - TTTTAG - 22 - A
	1768422 (-)	SPV_1769	TTTAAG - 5 - TTTAAC - 20 - A
	1907921 (-)[γ]	*malQ* (SPV_1933)	TTTAAA - 5 - TTTAAG - 23 - G
	2044340 (+)	*htrA* (SPV_2068)	TTTAAG - 5 - ATTAAG - 22 - A
		**Consensus CiaR**	**TTTAAG - 5 - TTTAAG - 22 - TSS**
	23967 (+)	*ccnC* (SPV_2078)	TTTAAG - 5 - TTTAAG - 22 - G
	98483 (+)	SPV_0098	TAACAG - 5 - TTTAAG - 23 - A
	788851 (+)	SPV_0775	TTTAAG - 5 - TTTAAA - 22 - A
No diff. espression[β]	212734 (+)	*ccnE* (SPV_2125)	TTTAAG - 5 - TTTAAG - 22 - A
	231599 (+)	*ccnA* (SPV_2129)	CTTAAG - 5 - TTTAAG - 22 - A
	231787 (+)	*ccnB*(SPV_2130)	GTTAAG - 5 - TTTAAG - 22 - A
	1159217 (-)[γ]	*tarI*(SPV_1127)	TTTAAG - 5 - TTTAAG - 21 - A
Rev. compl.	263684 (-)[δ]	*manL* (SPV_0264)	TTTAAG - 5 - TTTAAG ACTG TTTAAG
	487564 (+)[δ]	*rimP* (SPV_0478)	T - 59 - TTTAGG - 6 - CTTAAA
	1982193 (-)[δ]	*dltX* (SPV_2006)	T - 60 - TTTCAG - 5 - TTTAAG

Figure S2. CiaR-binding sequences on the *S. pneumoniae* D39V genome. Consensus sequence was determined as described in **Materials and Methods**. HC: high-confidence. [α]First gene both annotated in D39V and D39W (4), if available. [β]See **Table 4**. [γ]The *tarIJ-licABC* and *malQP* operons are each under control of two different TSSs, one of which is regulated by CiaR. [δ]Displayed sequences are reverse complementary to the transcription direction. In the case of *manLMN*, this leads to CiaR-mediated repression rather than activation.

	TSS	First gene[α]	
HC VraR regulon	156057 (+)	llmg_0165 (*L. lactis*)	TCAGTCGAAAGTCTTA - 32 - A
	159668 (+)	llmg_0169 (*L. lactis*)	TCAGTCAAAAGACTGA - 34 - A
	1074450 (+)	llmg_1115 (*L. lactis*)	TCAGCCCTAAGTATGA - 33 - A
	1124053 (+)	llmg_1155 (*L. lactis*)	TACATCTCAAGTCTGA - 33 - A
	1627838 (-)	llmg_1650 (*L. lactis*)	TAAGTCTTAAGTCCGA - 34 - A
	2125753 (-)	llmg_2164 (*L. lactis*)	TCAGCCCAAAGGCTGA - 34 - A
	183663 (+)[β]	*spxA2* (SPV_0178)	TAGGTCGCAAGTCGGA - 34 - A
	354945 (+)	*vraT* (SPV_0350)	TCCATCCTCAGACTGA - 33 - G
	820339 (+)	SPV_0803	TCAGCCTGAGGACTGA - 34 - A
		**Consensus VraR**	TMMGYCNNAAGWCTGA - 34 - TSS
	467922 (+)[γ]	*hrcA* (SPV_0458)	TAGGTCTTCAGATTGA - 81 - A
No diff. espression	Unknown[δ]	SPV_1057	TCGAACTTATGAAGAA
	Unknown[ε]	SPV_1160	TTATTCCAAAGACTCA

Figure S3. VraR-binding sequences on the *S. pneumoniae* D39V and *L. lactis* genome. Consensus sequence was determined as described in **Materials and Methods**. Multiple possible nucleotides are indicated, according to IUPAC nomenclature, by Y (C/T), W (A/T), M (A/C) or N (any). HC: high-confidence. [α]First gene in *L. lactis* or first gene both annotated in D39V and D39W (4), if available. [β]Gene *spxA2* is under control of two different TSSs, one of which is regulated by VraR. [γ]The *hrcA* operon is coregulated by HrcA and VraR, possibly accounting for the different spacing to the TSS. [δ]Reported site (11) is located downstream of SPV_1057, inside a transposon sequence. [ε]Distance between reported site (11) and annotated start codon of SPV_1160 is 24 nucleotides. Moreover, SPV_1160 is a 5'-truncated pseudogene.

Supplemental references

1. Langfelder P, Horvath S. 2008. WGCNA: an R package for weighted correlation network analysis. BMC Bioinformatics 9:559.

2. Eldholm V, Gutt B, Johnsborg O, Brückner R, Maurer P, Hakenbeck R, Mascher T, Håvarstein LS. 2010. The pneumococcal cell envelope stress-sensing system LiaFSR is activated by murein hydrolases and lipid II-interacting antibiotics. J Bacteriol 192:1761–1773.

3. Kery MB, Feldman M, Livny J, Tjaden B. 2014. TargetRNA2: identifying targets of small regulatory RNAs in bacteria. Nucleic Acids Res 42:W124-129.

4. Lanie JA, Ng W-L, Kazmierczak KM, Andrzejewski TM, Davidsen TM, Wayne KJ, Tettelin H, Glass JI, Winkler ME. 2007. Genome sequence of Avery's virulent serotype 2 strain D39 of *Streptococcus pneumoniae* and comparison with that of unencapsulated laboratory strain R6. J Bacteriol 189:38–51.

5. Meyer MM, Roth A, Chervin SM, Garcia GA, Breaker RR. 2008. Confirmation of a second natural preQ$_1$ aptamer class in Streptococcaceae bacteria. RNA N Y N 14:685–695.

6. Narberhaus F. 1999. Negative regulation of bacterial heat shock genes. Mol Microbiol 31:1–8.

7. Derré I, Rapoport G, Msadek T. 1999. CtsR, a novel regulator of stress and heat shock response, controls *clp* and molecular chaperone gene expression in gram-positive bacteria. Mol Microbiol 31:117–131.

8. Novichkov PS, Kazakov AE, Ravcheev DA, Leyn SA, Kovaleva GY, Sutormin RA, Kazanov MD, Riehl W, Arkin AP, Dubchak I, Rodionov DA. 2013. RegPrecise 3.0--a resource for genome-scale exploration of transcriptional regulation in bacteria. BMC Genomics 14:745.

9. Slager J, Aprianto R, Veening J-W. 2018. Deep genome annotation of the opportunistic human pathogen *Streptococcus pneumoniae* D39. Nucleic Acids Res 46:9971–9989.

10. Bonner ER, D'Elia JN, Billips BK, Switzer RL. 2001. Molecular recognition of *pyr* mRNA by the *Bacillus subtilis* attenuation regulatory protein PyrR. Nucleic Acids Res 29:4851–4865.

11. Love MI, Huber W, Anders S. 2014. Moderated estimation of fold change and dispersion for RNA-seq data with DESeq2. Genome Biol 15:550.
